# α-ketoglutarate dehydrogenase inhibition counteracts breast cancer-associated lung metastasis

**DOI:** 10.1038/s41419-018-0802-8

**Published:** 2018-07-09

**Authors:** Sandra Atlante, Alessia Visintin, Elisabetta Marini, Matteo Savoia, Chiara Dianzani, Marta Giorgis, Duran Sürün, Federica Maione, Frank Schnütgen, Antonella Farsetti, Andreas M. Zeiher, Massimo Bertinaria, Enrico Giraudo, Francesco Spallotta, Chiara Cencioni, Carlo Gaetano

**Affiliations:** 1 Division of Cardiovascular Epigenetics, Department of Cardiology, Goethe University, 60596 Frankfurt am Main, Germany; 2 Laboratory of Transgenic Mouse Models, Candiolo Cancer Institute – FPO, IRCCS, Candiolo, Italy; 3 Dipartimento di Scienza e Tecnologia del Farmaco, Università degli Studi di Torino, 10125 Torino, Italy; 4 Department of Medicine, Hematology/Oncology, Goethe University, 60596 Frankfurt, Germany; 5 Istituto di Biologia Cellulare e Neurobiologia (IBCN), Consiglio Nazionale delle Ricerche (CNR), 00143 Roma, Italy; 6 Internal Medicine Clinic III, Department of Cardiology, Goethe University, Frankfurt am Main, Germany; 7 Laboratorio di Epigenetica, Istituti Clinici Scientifici Maugeri, Via Maugeri 4, 27100 Pavia, Italy

## Abstract

Metastasis formation requires active energy production and is regulated at multiple levels by mitochondrial metabolism. The hyperactive metabolism of cancer cells supports their extreme adaptability and plasticity and facilitates resistance to common anticancer therapies. In spite the potential relevance of a metastasis metabolic control therapy, so far, limited experience is available in this direction. Here, we evaluated the effect of the recently described α-ketoglutarate dehydrogenase (KGDH) inhibitor, (S)-2-[(2,6-dichlorobenzoyl) amino] succinic acid (AA6), in an orthotopic mouse model of breast cancer 4T1 and in other human breast cancer cell lines. In all conditions, AA6 altered Krebs cycle causing intracellular α-ketoglutarate (α-KG) accumulation. Consequently, the activity of the α-KG-dependent epigenetic enzymes, including the DNA demethylation ten-eleven translocation translocation hydroxylases (TETs), was increased. In mice, AA6 injection reduced metastasis formation and increased 5hmC levels in primary tumours. Moreover, in vitro and in vivo treatment with AA6 determined an α-KG accumulation paralleled by an enhanced production of nitric oxide (NO). This epigenetically remodelled metabolic environment efficiently counteracted the initiating steps of tumour invasion inhibiting the epithelial-to-mesenchymal transition (EMT). Mechanistically, AA6 treatment could be linked to upregulation of the NO-sensitive anti-metastatic miRNA 200 family and down-modulation of EMT-associated transcription factor Zeb1 and its CtBP1 cofactor. This scenario led to a decrease of the matrix metalloproteinase 3 (MMP3) and to an impairment of 4T1 aggressiveness. Overall, our data suggest that AA6 determines an α-KG-dependent epigenetic regulation of the TET–miR200–Zeb1/CtBP1–MMP3 axis providing an anti-metastatic effect in a mouse model of breast cancer-associated metastasis.

## Introduction

For its high yearly incidence, mortality and morbidity, breast cancer is a developing threat women face worldwide^1,2^. The disease is extremely heterogeneous^3^ and characterised by about 20% incidence of metastasization^2^ mainly in bone, distant soft tissue and lung^4,5^. Despite the remarkable progresses in prevention and patient care and the scientific community effort to elucidate the molecular mechanism underpinning aetiology and development of breast cancer, the request of effective anti-metastatic therapies remains open.

Recently, a broad interest pointed to cancer metabolism as a promising target to develop new therapeutic approaches. Cancer cells are characterised by a hyperactive metabolism and adaptability to nutrient deprivation^6^. Indeed, enhanced glycolysis and/or oxidative phosphorylation conferred to drugs interfering with metabolism, including the tricarboxylic acid (TCA) cycle, promising therapeutic potential interest, although the possibility to elicit adverse effects needs to be carefully evaluated^7–10^. TCA helps cancer to develop its adaptability in consequence of the intrinsic ability to adjust metabolic fluxes according to resource availability. Further, metabolites produced during TCA cycle dramatically affect tumour cell epigenetic landscape^11–13^. In this light, TCA cycle relevance is validated by several specific cancer-associated mutations occurring into the coding sequence of its enzymes^14,15^. In mitochondria, the α-ketoglutarate dehydrogenase complex (KGDH), a key control TCA enzyme, catalyses the oxidative decarboxylation of α-ketoglutarate (α-KG) to succinyl-CoA exploiting the reduction of NAD^+^ to NADH^12,16–18^. Its enzymatic activity relies on the availability of ATP, inorganic phosphate, and NAD^+^ produced by glycolysis and respiratory chain controlling the mitochondrial redox status, the metabolite flux and many different signalling pathways, including amino acid synthesis^15,19,20^. KGDH is one of the mitochondrial enzymes most sensitive to tumour micro-environmental changes and plays a role in the cancer adaptive metabolic response^6,21^. Therefore, it is envisaged that drugs targeting this enzymatic complex might show interesting anti-cancer properties.

DNA hypermethylation is an intrinsic feature of cancer genetic landscape^22–24^ possibly due to ten-eleven translocation hydroxylase (TET) activity alterations^25^, which have been associated with worse prognosis^22–24^. Commonly, in cancer, the reduced DNA demethylation associates with specific mutations or decreased expression of TET encoding genes, as well as with diminished α-KG intracellular levels occurring upon its replacement with the oncometabolite D-2-hydroxyglutarate^25–28^. α-KG not only fuels energetic and anabolic routes into the mitochondrion but regulates also demethylation of DNA and histones, acting as cofactor for all dioxygenases including TETs and Lysine demethylases (KDMs)^29–31^. Of interest, in a metabolically compromised environment, KGDH inhibition increased α-KG level restoring the epi-metabolic control on the DNA demethylation cycle^32^.

TET activity is particularly relevant to counteract breast cancer progression by suppression of mechanisms associated with the metastatic process^33–35^. In this context, TET proteins de-repress the expression of tissue inhibitors of metalloproteinases (TIMP 2 and 3)^36^ and of anti-metastatic miRNAs, such as miR-200 family members, demethylating their promoter regions^35^.

The miR-200 family consists of five members organised in two different clusters according to chromosomal location. Mouse chromosome 4 and 6 give rise to two polycistronic transcripts encoding for cluster 1 (miR-200b, miR-200a and miR-429) and cluster 2 (miR-200c and miR-141) respectively^37^. In breast cancer they hinder both epithelial-to-mesenchymal transition (EMT), the initiating step of tumour invasion, and metastatic cancer stem cell function^37–39^. Most of miR-200 tumour suppressor activity is obtained by direct targeting of the two zinc-finger E-box binding homeobox members Zeb1 and Zeb2^40–42^. This family of transcription factors have been defined as the master inducer/regulator of EMT since they directly inhibit the cell-cell adhesion molecule E-cadherin enhancing cell motility^40–42^.

Although metabolic alterations, inefficient DNA demethylation and unbalanced miR-200/Zeb circuitry have been well defined as crucial steps along metastatic progression, the presence of a functional link among all these elements has not been thoroughly investigated yet. In the present work, we took advantage from the properties of a novel compound, the (S)-2-[(2,6-dichlorobenzoyl)amino]succinic acid (AA6), able to inhibit KGDH activity, to increase cellular α-KG levels and to restore the epi-metabolic control upon DNA demethylation cycle^32^. Here, we investigated AA6 properties as potential anti-metastatic drug in a spontaneous lung metastasis mouse model of breast cancer.

## Results

### The KGDH inhibitor AA6 prevents lung metastasis formation in 4T1 mouse model of breast cancer

**Fig. 1:**
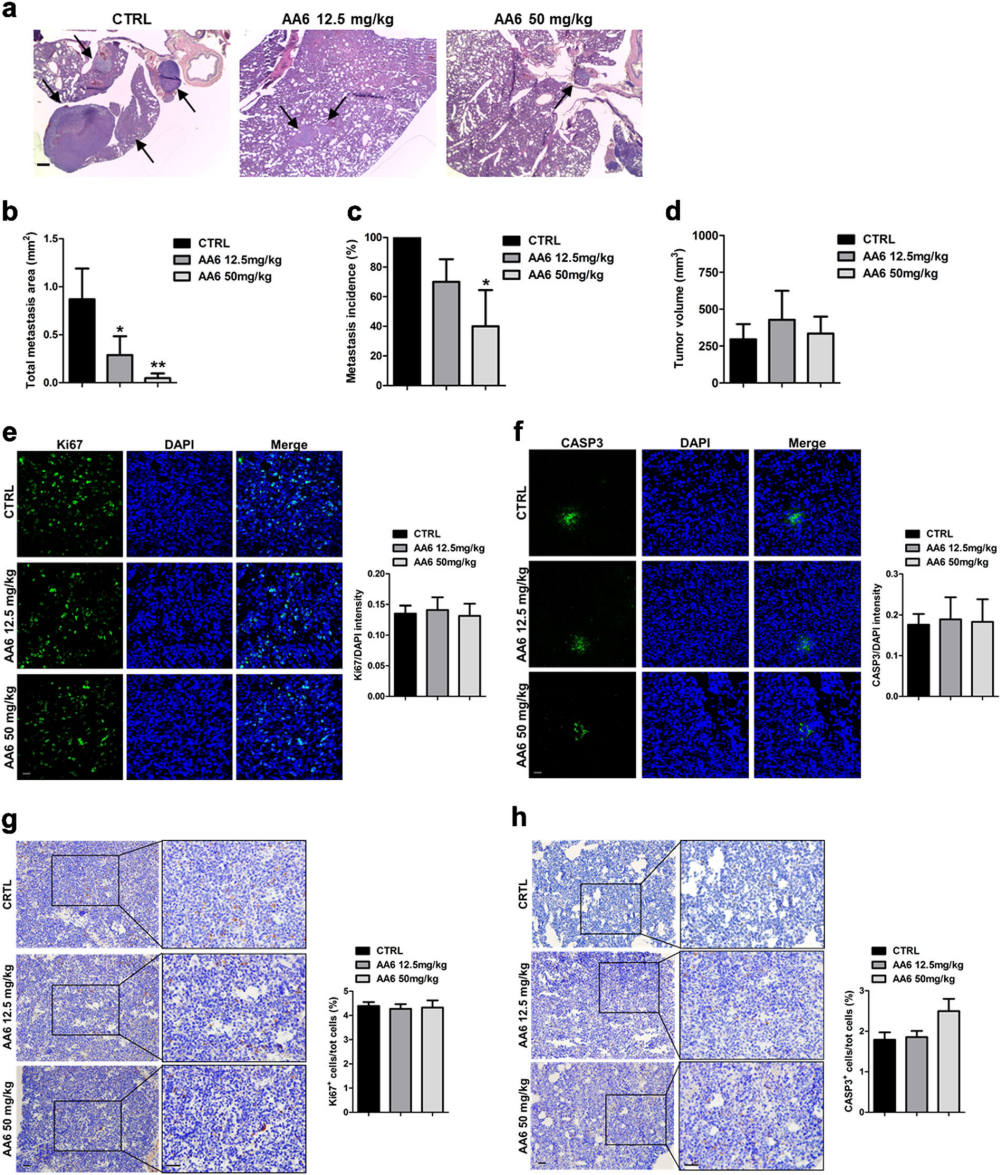
AA6 prevents 4T1 cell lung metastasis formation. **a** Hematoxylin and eosin staining: arrows represent lung metastasis reduction after 3 weeks of treatment of 4T1 orthotopic mouse model of breast cancer with AA6 (12.5 mg/kg *n* = 10; 50 mg/kg *n* = 5), compared to controls; *n* = 10. Scale bar 50 μm. **b–d** The graph shows the measured metastasis area in AA6 treated mice (12.5–50 mg/kg; grey bars) compared to controls (black bars) (**b**) and the percentage of metastasis incidence analysed by Mantel-Cox Test (*p = 0.0082) (**c**); no difference was observed in the primary - tumour volume (**d**). **e**, **f** Representative confocal images (left panels) and relative densitometry (right panels) showing cell proliferation (Ki67) (**e**) and apoptosis (CASP3) (**f**) in AA6 treated mice primary tumour (12.5–50 mg/kg; grey bars) compared to controls (black bars). Samples were probed by an anti-Ki67 antibody (green; monoclonal), anti-CASP3 (green; monoclonal) and counterstained by DAPI (blue). Scale bar 50 μm; control *n* = 10; 12.5 mg/kg *n* = 10; 50 mg/kg *n* = 5. **g**, **h** Representative immunohistochemistry images (left panels) and relative quantification (right panels) showing cell proliferation (Ki67) (**g**) and apoptosis (CASP-3) (**h**) in AA6 treated mice primary tumour (grey bars) compared to controls (black bars). Samples were probed by an anti-Ki67 antibody (monoclonal), anti-CASP3 (monoclonal) and counterstained by hematoxylin. Scale bar 50 μm; control *n* = 10; 12.5 mg/kg *n* = 10; 50 mg/kg *n* = 5. Data are presented as mean ± SE; *p < 0.0332, **p < 0.0021, ***p < 0.0002 vs controls. Data were analysed by non-parametric Mann–Whitney test

**Fig. 2:**
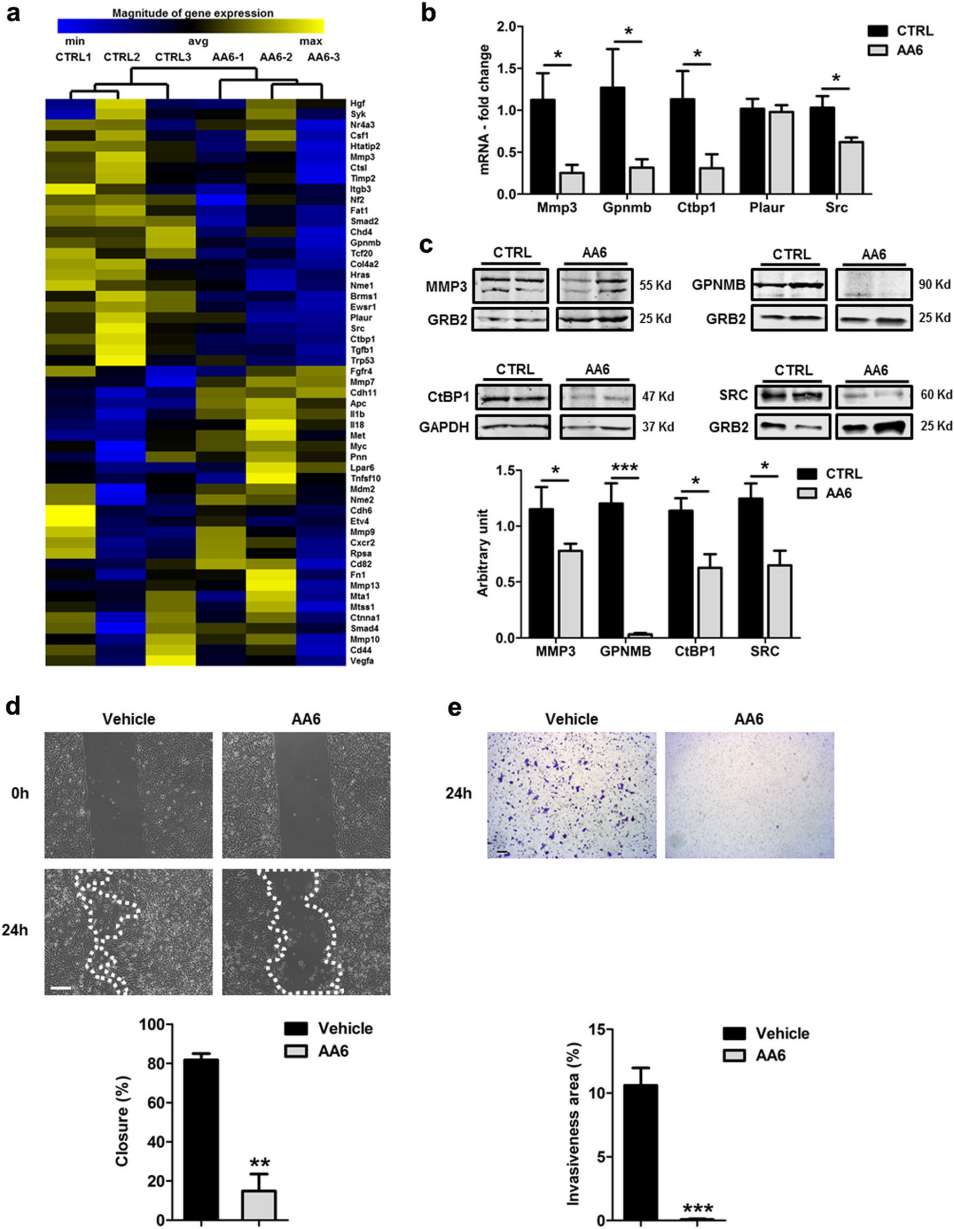
AA6 administration decreases metastasis-associated transcripts and interferes with 4T1 cells migration. **a** Heatmap showing the 53 most differentially regulated genes in tumour mass derived from AA6 injected mice (50 mg/kg), or untreated mice; *n* = 3 each group. Yellow and blue represent over- and under-expressed genes, respectively. **b** mRNA expression analysis of Matrix metallopeptidase 3 (Mmp3), Glycoprotein transmembrane non-metastatic B (Gpnmb), C-terminal binding protein 1 (Ctbp1), Plasminogen activator, urokinase receptor (Plaur) and Rous sarcoma oncogene (Src) genes in AA6 injected mice (50 mg/kg; grey bars) and control mice (black bars); *n* = 5. **c** Representative western blot (upper panels) and relative densitometry (lower panel) of MMP3, GPNMB, CtBP1 and SRC protein levels in AA6 (50 mg/kg; grey bars) treated mice compared to controls (black bars). GRB2 and GAPDH were used as loading controls; *n* = 5 each group. **d** Representative phase contrast microscopy images (upper panel) depicting 4T1 cells motility after 24 h treatment with AA6 (50 µM) or vehicle alone; the graph (lower panel) shows the percentage of closure in 4T1 cells after 24 h treatment with AA6 (50 µM; grey bar) or vehicle (black bar). Scale bar 100 μm; *n* = 5 each group. **e** Representative pictures (upper panel) showing 4T1 cell invasiveness after AA6 (50 µM) treatment versus vehicle; the graphs (lower panel) represent migrated cells counted after 24 h treatment with AA6 (50 µM; grey bar) or vehicle alone (black bar). Scale bar 50 μm; *n* = 3. Data are presented as mean ± SE; *p < 0.05, **p < 0.005, ***p < 0.0005 vs controls. Data were analysed by two-way ANOVA and non-parametric two-tailed paired Student’s *t*-test

The KGDH is a TCA cycle mitochondrial enzyme whose activity can be inhibited by (S)-2-[(2,6-dichlorobenzoyl)amino]succinic acid (AA6)^32^. In an attempt to understand whether its inhibition might interfere with tumour progression, we administered two doses of AA6 (12.5 mg/kg and 50 mg/kg) in the 4T1 orthotopic mouse model of breast cancer^43^. Interestingly, AA6 reduced the area of lung metastasis in a dose-dependent manner without apparently affecting growth of the primary tumour (Fig. 1a, b, d). Further the treatment of 4T1 mice with the dose of 50 mg/kg significantly decreased the incidence of lung metastasis (Fig. 1c). Analysis of Ki67, a marker of proliferating cells, and cleaved caspase 3 (Casp3), a marker of apoptosis, into the primary tumour of untreated and AA6 treated mice revealed no difference between the two groups. Similarly, we did not detect any significant differences in proliferation or apoptosis in lung metastasis (Fig. 1e–h). These data suggest that AA6 does not directly interfere with metastasis growth but rather could induce a delay of the metastatic process. To investigate the anti-metastatic effect of AA6, we studied the expression of a selected panel of genes associated with tumour progression and metastasis. This analysis revealed that some crucial metastatic genes were negatively modulated by AA6 (Fig. 2a and Supplemental table I). Further analyses confirmed that AA6 repressed, both at mRNA and protein level, extracellular matrix proteases (e.g. Mmp3), cell adhesion molecules (e.g. Gpnmb), and transcription factors associated to cancer progression (e.g. Ctbp1) with a minor effect on cell proliferation genes (e.g. Plaur and Src) (Fig. 2b, c). In agreement, 4T1 cells treated with AA6 for 24 h (h) showed reduced migration (Suppl. Figure 1a, b), adhesion to endothelium (Suppl. Figure 1c) and invasion capacity (Suppl. Figure 1d). In these experiments, the most important effects were observed at 50 µM AA6, with a tumour cell migration impairment/inhibition higher than 80% (Fig. 2d), in parallel to a significant inhibition of invasiveness (Fig. 2e). In agreement with in vivo observations, 50 µM AA6 did not affect survival and proliferation of 4T1 cells (Suppl. Figure 2). Interestingly, similar results were obtained in two different human breast cell lines: the African American human cell line CRL-2335 and the Caucasian human cell line MDA-MB-231. CRL-2335 cell line is a recognized model of basal-like breast carcinoma, one of the most aggressive and deadly carcinoma sub-type characterized by poor clinical outcomes. Of note, CRL-2335 cells are negative for the expression of human epidermal growth factor receptor 2 (Her2-neu), Oestrogen Receptor (ER), and Progesterone Receptor (PR) and positive for the expression of basal-like markers, epidermal growth factor (EGFR), and cytokeratin 5/6 (ck 5/6)^44^. Conversely, the MDA-MB-231 cell line represents one of the most commonly used breast cancer highly aggressive, invasive and poorly differentiated triple-negative (Her2-neu, ER, and PR negative) in vitro model^45,46^. Remarkably, AA6 down-modulated most of the metastasis-associated genes in both human breast cell lines (Suppl. Figure 3a, b; Suppl. Figure 4a, b). Further, AA6 reduced serum-stimulated chemo-attraction (Suppl. Figure 3c; Suppl. Figure 4c) and adhesion to TNF-α-activated endothelium (Suppl. Figure 3d; Suppl. Figure 4d) without affecting viability (Suppl. Figure 3e; Suppl. Figure 4e), survival and proliferation (Suppl. Figure 3f; Suppl. Figure 4f). Hence, AA6 treatment affected tumorigenic functions of 4T1, CRL-2335 and MDA-MB-231 cells without altering their vital functions. Taken together these results foster AA6 as an anti-metastatic compound active both in vivo and in vitro.

### KGDH inhibition increases α-KG levels leading to DNA demethylation and impaired cell migration

**Fig. 3:**
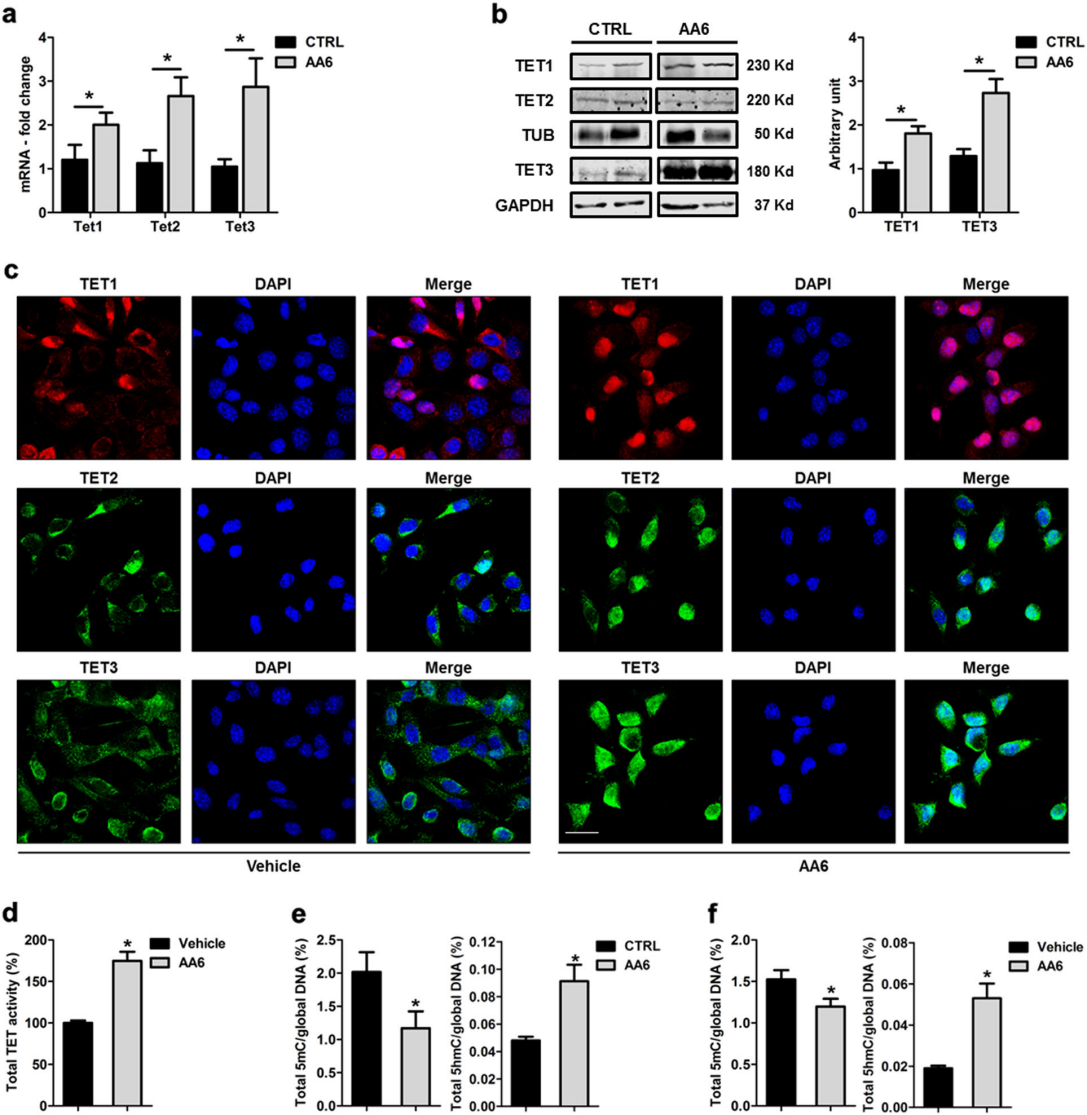
KGDH inhibition increases TET expression and modulates 5mC/5hmC global levels both in vivo and in vitro. **a** Ten-eleven translocation hydroxylases (Tet) -1, 2, 3 mRNA expression levels in AA6 injected mice (50 mg/kg; grey bars) and control mice (black bars); *n* = 5. **b** Representative western blot (left panel) and relative densitometry (right panel; *n* = 4) of TET1, 2, 3 in AA6 (50 mg/kg; grey bars) treated mice compared to controls (black bars). α-tubulin and Glyceraldehyde 3-phosphate dehydrogenase (GAPDH) were used as a loading controls. **c** Representative confocal images depicting the intracellular content of TET1, 2, 3 enzymes in 4T1 cells treated with AA6 (50 µM) or vehicle alone. Cells were probed by an anti-TET1 antibody (red; monoclonal), TET2 (green; polyclonal), TET3 (green; polyclonal) and counterstained by DAPI (blue). Scale bar 25 μm; *n* = 3. **d** TET activity quantification performed in 4T1 cells treated with AA6 (50 µM; grey bar) for 48 h indicated as percentage versus vehicle-treated cells (black bar); *n* = 3. **e** Quantification of 5mC (left panel) and 5hmC (right panel) global levels in 4T1-injected mice after AA6 administration (50 mg/kg; grey bars) compared to untreated mice (black bars); *n* = 5 each group. **f** Quantification of 5mC (left panel) and 5hmC (right panel) global levels in 4T1 cells exposed to AA6 (50 µM; grey bars) for 48 h indicated as fold-change versus vehicle-treated cells (black bars); *n* = 3 each group. Data are presented as mean ± SE; *p < 0.05 vs controls. Data were analysed by two-way ANOVA and non-parametric two-tailed paired Student’s *t*-test

**Fig. 4:**
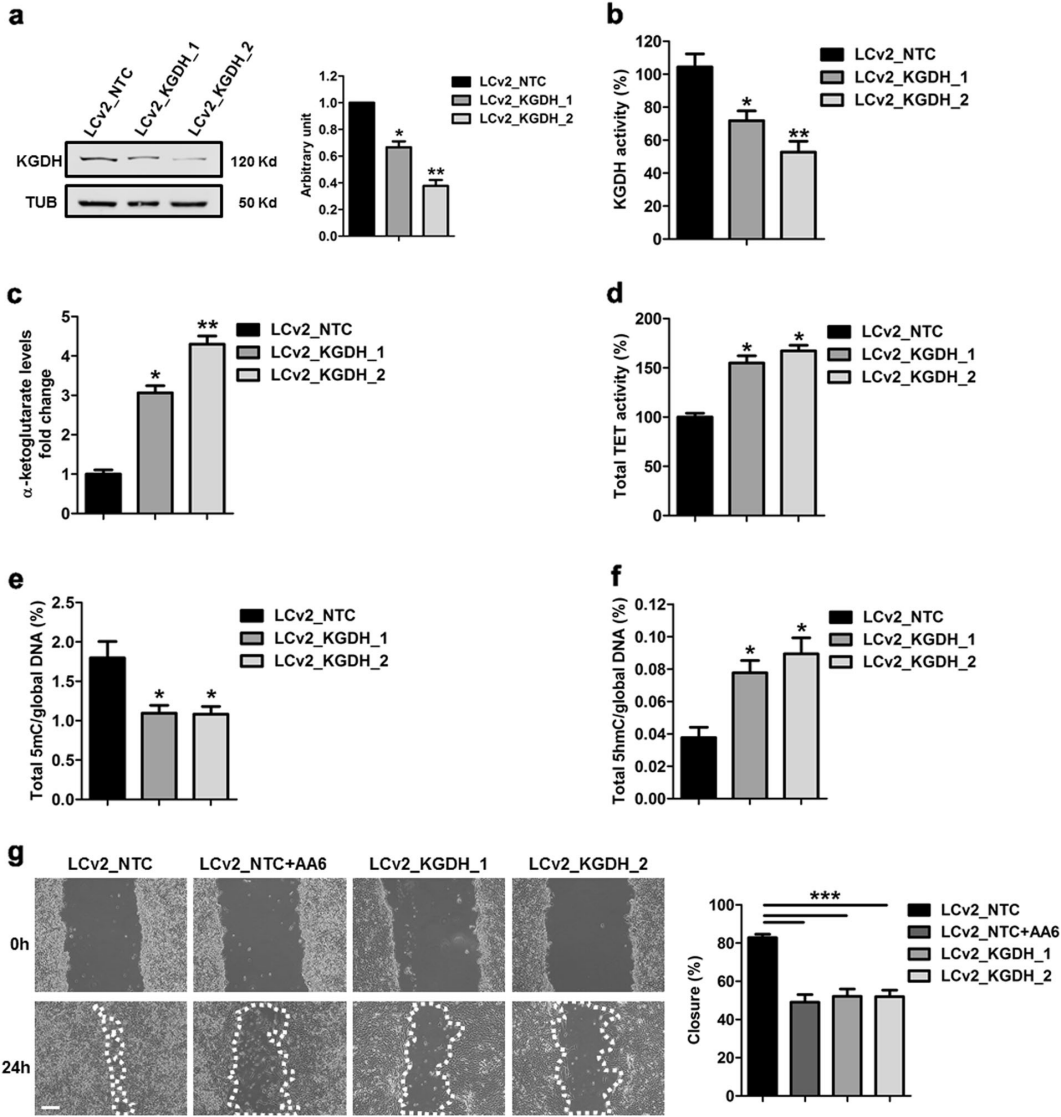
CRISPR/Cas9 KGDH inactivation increases α-KG levels, TET activity and global 5hmC and interferes with 4T1 cell line biological properties. **a** Representative WB (left panel) and relative densitometry (right panel) of KGDH protein levels in 4T1 cells after CRISPR/Cas9 inactivation of KGDH (LCv2_KGDH_1 and LCv2_KGDH_2) compared to control vector (LCv2_NTC). α-tubulin was used as a loading control; *n* = 5. **b** KGDH activity and **c** α-KG level quantification of LCv2_NTC- (black bars), LCv2_KGDH_1- (dark grey bars) and LCv2_KGDH_2- (light grey bars) 4T1 cells; *n* = 3 each group. **d** TET activity quantification performed in LCv2_KGDH_1- (dark grey bar) and LCv2_KGDH_2- (light grey bar) 4T1 cells compared to LCv2_NTC (black bar); *n* = 3. **e** Global 5mC and **f** 5hmC levels in 4T1 cells after CRISPR/Cas9 inactivation of KGDH (LCv2_KGDH_1 and LCv2_KGDH_2; grey bars) compared to control vector (LCv2_NTC; black bars); *n* = 3 each group. **g** Representative phase contrast microscopy images (left panel) and relative percentage of closure measurements (right panel) showing 4T1 cells motility after CRISPR/Cas9 inactivation of KGDH (LCv2_KGDH_1; medium grey bar and LCv2_KGDH_2; light grey bar) compared to control vector (LCv2_NTC; black bar) in the presence or absence of AA6 (50 µM; dark grey bars). Scale bar 100 μm; *n* = 3 each condition. Data are presented as means ± SE; *p < 0.05, **p < 0.005, ***p < 0.0005 vs controls. Data were analysed by non-parametric two-tailed paired Student’s *t*-test

In agreement with prior data obtained from non-cancer related dysmetabolic models^32^, also in breast cancer AA6 inhibited KGDH enzymatic activity (Suppl. Figure 5a) leading to intracellular α-KG level increase (Suppl. Figure 5b). In this chemically determined α-KG-enriched environment, we observed an increase in TET protein expression both in vivo (Fig. 3a, b) and in vitro (Fig. 3c and Suppl. Figure 5c) paralleled by a higher total TET enzymatic activity (Fig. 3d). Interestingly, in 4T1 cells, confocal analysis of TET proteins revealed that AA6 treatment rescued the predominant cancer-associated extra-nuclear localisation of these proteins^47,48^ (Fig. 3c). TET1 and TET3 re-localisation into the nucleus was further confirmed by the biochemical analysis of nucleus/cytoplasm fractions (Suppl. Figure 5d). Remarkably, the intra-nuclear TET re-localisation was paralleled by a global reduction of DNA 5-methyl cytosine (5mC) and a relative increase in the content of 5-hydroxymethyl cytosine (5hmC) both in vivo and in vitro (Fig. 3e, f). Similar results were observed in CRL-2335 cells and MDA-MB-231 cells upon AA6 treatment (Suppl. Figure 3g; Suppl. Figure 4g). In order to investigate AA6 molecular mechanism, KGDH expression was knocked-down (KD) in 4T1 cells by CRISPR/Cas9 technology (Fig. 4a). KGDH expression KD significantly affected its enzymatic activity (Fig. 4b) leading to a relative accumulation of intracellular α-KG (Fig. 4c). Of note, CRISPR/Cas9 vector 2 was more efficient than vector 1 eliciting a higher increase of α-KG levels. AA6 effect on total TET enzymatic activity as well as on 5mC and 5hmC was reproduced in 4T1 KGDH KD cells (Fig. 4d–f). Remarkably, the partial inhibition of KGDH impaired 4T1 cell migration (Fig. 4g) without altering cell viability and proliferation (Suppl. Figure 6a-c). All these evidences suggested that the KGDH complex might function as a key metabolic enzyme during metastatic progression and that AA6 counteracts this process.

### AA6 stimulates the endogenous synthesis of nitric oxide

**Fig. 5:**
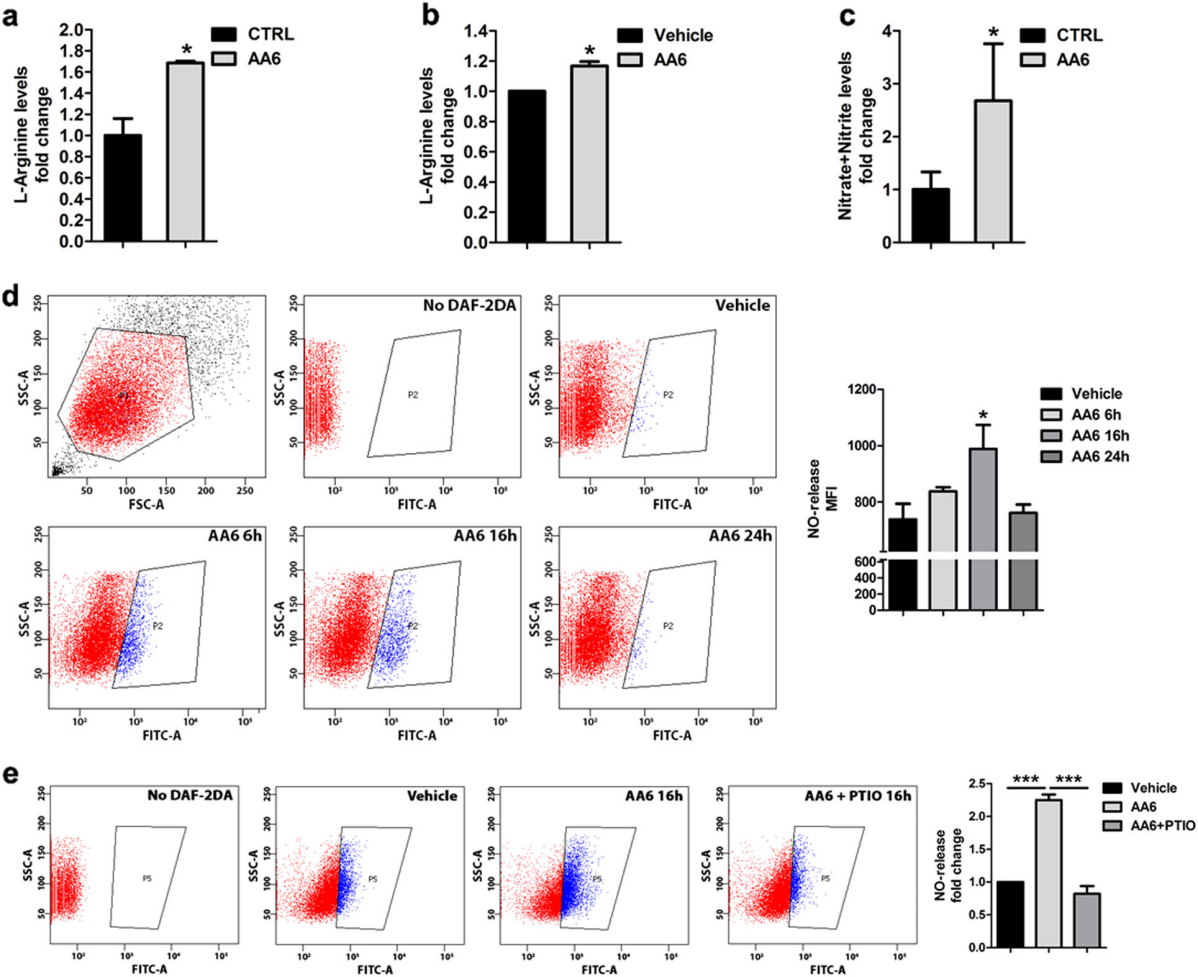
AA6 increases nitric oxide-release in 4T1 cells. **a** L-Arginine levels in 4T1-injected mice after AA6 administration (50 mg/kg; grey bar) compared to untreated mice (black bar); *n* = 3. **b** L-Arginine levels in 4T1 cells after 16 h of treatment with AA6 (50 µM; grey bar) or vehicle alone (black bar); *n* = 3. **c** Nitrate and nitrite quantification in 4T1-injected mice after AA6 administration (50 mg/kg; grey bar) compared to untreated mice (black bar); *n* = 3. **d** Representative dot plot (left panel) and relative densitometry (right panel) showing NO-release evaluation by FACS analysis of DAF-2DA-stained 4T1 cells, after 6, 16, and 24 h of treatment with AA6 (50 µM; grey bars), expressed as fold change to vehicle-treated cells (black bar); the gated cell populations (blue, P5) indicate DAF-2T-positive cells for each condition, negative controls were in absence of DAF-2D; *n* = 3 each group. **e** Representative dot plot (left panel) and relative densitometry (right panel) showing NO-release evaluation by FACS analysis of DAF-2 DA stained 4T1 cells after 16 h of treatment with vehicle only (black bar), AA6 (50 µM; light grey bar) and AA6 (50 µM) + PTIO (100 µM; dark grey bar); *n* = 4 each condition. Data are presented as mean ± SE; *p < 0.05, ***p < 0.0005 vs controls. Data were analysed by one-way ANOVA and non-parametric two-tailed paired Student’s *t*-test

Taking under consideration the impact that α-KG fluctuations might have on different intracellular pathways, we evaluated the involvement of additional mechanisms in the anti-metastatic response to AA6. α-KG is a relevant precursor of ornithine, a non-proteinogenic amino acid, which leads to L-arginine (L-Arg) synthesis through the urea cycle. As a substrate, L-Arg contributes to NO synthases (NOS) activation fostering the endogenous production of NO^49^. Recently, NO-donors have been proposed as promising therapeutic options for breast, liver and skin cancers^50^ and, in this perspective, we reasoned that the anti-metastatic role of AA6 might be not only associated to its modulation of mitochondrial metabolism and TET activation but also to an effect on endogenous NO synthesis. To explore this possibility, we investigated AA6 effect on the α-KG/L-Arg/NO axis. A slight but significant increase of L-Arg content was observed both in vivo (Fig. 5a) and in vitro (Fig. 5b) either upon AA6 treatment or after KGDH KD (Suppl. Figure 6d). Interestingly, an increase in the total amount of nitrates and nitrites, conceivably by-products of NO synthesis, was detected in the tumorigenic tissue of AA6 treated mice (Fig. 5c). In agreement with this observation, after 16 h of AA6 in vitro treatment, increased NO levels were detected in 4T1 cells as determined by the FACS analysis of signals generated by the fluorescent indicator 4,5-diaminofluorescein-2 diacetate (DAF-2DA; Fig. 5d). The ability of AA6 to induce endogenous NO synthesis was verified by the addition of the NO scavenger 2-Phenyl-4,4,5,5-tetramethylimidazoline-1-oxyl 3-oxide (PTIO) to 4T1 cells treated with AA6. As expected, PTIO significantly reduced NO levels (Fig. 5e). Consistently, similar increase in NO production was observed in 4T1 KGDH KD cells (Suppl. Figure 6e). Taken together these data suggest that AA6 has the unprecedented property to activate NO synthesis possibly acting *via* enhancement of the metabolic/biosynthetic α-KG/L-Arg/NO axis.

### AA6 impairs the EMT process through activation of miR-200 family and Zeb1 down modulation

**Fig. 6:**
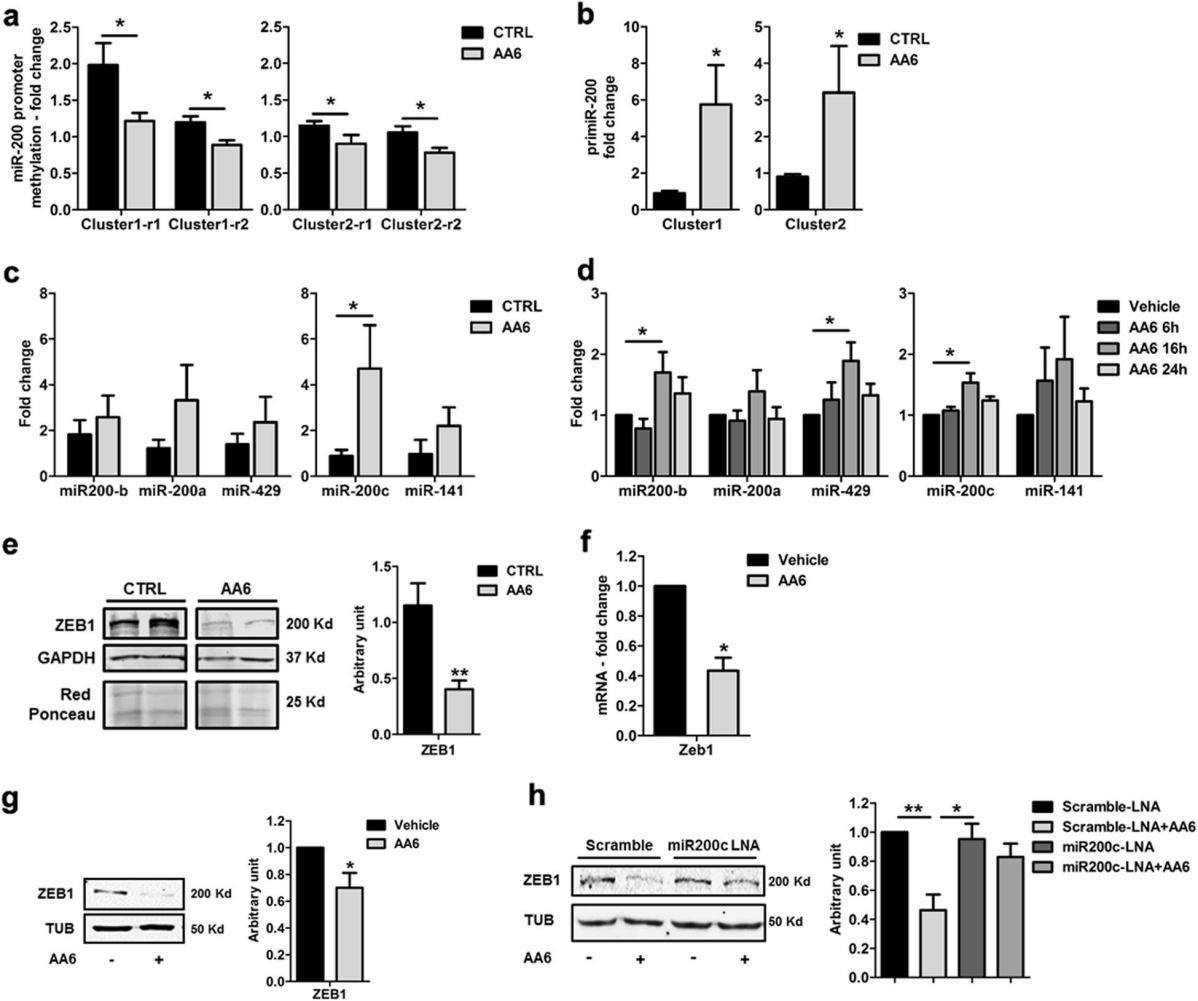
AA6 prevents metastasization targeting the TET–miR200–Zeb1/CtBP1–MMP3 axis. **a** Relative enrichment of 5mC in selected CCpGG sites of miR-200 family promoter regions for cluster 1 (left panel) and cluster 2 (right panel) in 4T1-injected mice DNA treated with AA6 (50 mg/kg; grey bars) versus control mice DNA (black bars); *n* = 5. **b** Pri-miR-200 cluster 1 (left panel) and cluster 2 (right panel) level of AA6 (50 mg/kg; grey bars) treated 4T1-injected mice expressed as fold-induction compared to untreated mice (black bars); *n* = 3. **c** Cluster 1 (miR-200b, miR-200a and miR-429; left panel) and cluster 2 (miR-200c and miR-141; right panel) expression in 4T1-injected mice treated with AA6 (50 mg/kg; grey bars), the graph represents average fold changes versus controls (black bars); *n* = 4. **d** Cluster 1 (miR-200b, miR-200a and miR-429; left panel) and cluster 2 (miR-200c and miR-141; right panel) expression in 4T1 cells treated with AA6 (50 µM; grey bars) for 6, 16, and 24 h, bar graphs represent average fold changes versus vehicle-treated cells (black bars); *n* = 4. **e** Representative WB (left panel) and relative densitometry (right panel; *n* = 5) of ZEB1 protein level in AA6 (50 mg/kg; grey bar) treated mice compared to controls (black bar). GAPDH and Red Ponceau were used as loading controls. **f**, **g** Zeb1 mRNA expression levels (**f**) and representative western blotting analysis of ZEB1 protein expression (**g**) in 4T1 cells exposed to AA6 (50 µM; grey bars) for 48 h indicated as fold-change versus vehicle-treated cells (black bars); the right panel shows the relative densitometry as fold-change versus vehicle. α-tubulin was used as loading control; *n* = 4. **h** Representative WB (left panel) and relative densitometry (right panel) of ZEB1 protein expression level in AA6 treated 4T1 cells compared to vehicle-treated cells after transfection either with scramble-LNA (vehicle: black bar; AA6 50 µM: light grey bar) or anti-miR-200c-LNA (vehicle: dark grey bar; AA6 50 µM: medium grey bar). α-tubulin was used as loading control; *n* = 4. Data are presented as mean ± SE; *p < 0.05, **p < 0.005 vs controls. Data were analysed by one and two-way ANOVA and non-parametric two-tailed paired Student’s *t*-test

It has been recently reported that miR-200 family expression is controlled by endogenous NO allowing for the mesendodermal differentiation of mouse embryonic stem cells^51,52^. miR-200 family is usually down-modulated during tumour progression. This phenomenon is believed to prevent the miR-200-dependent inhibition of: (i) EMT; (ii) cancer stem cell self-renewal/differentiation; (iii) chemoresistance^37–39^. Intriguingly, as miR-200 family presents CpG-rich sequences, a down-modulation mechanism used by cancer is the hypermethylation at the regulatory regions of both clusters to favour tumour formation and increase cell invasion ability^25,27,35^. To understand whether AA6 treatment might have an effect on the tumour suppressor miR-200 family we investigated DNA methylation at miR-200 gene loci and their response to endogenous NO production in 4T1 cells. Methylation analysis revealed that AA6 significantly reduced 5mC levels in two different regulatory regions of both miR-200 cluster 1 and 2 (Fig. 6a). The methylation reduction allowed miR-200 family transcription in vivo (Fig. 6b, c) and in vitro (Fig. 6d) as shown by pri-miR and miR parallel expression analysis. AA6 ability to positively regulate NO production and miR-200 family expression prompted us to investigate other players of the EMT process, such as the transcriptional repressor Zeb1, a direct target of miR-200s.

Zeb1, a zinc-finger homeodomain transcription factor, actively facilitates EMT by transcriptional inhibition of the cell–cell adhesion molecule E-cadherin, a hallmark of the initiating step of cancer metastasis. AA6 administration in mice injected with 4T1 cells into the mammary gland significantly reduced Zeb1 protein level (Fig. 6e). The same result was obtained in 4T1, CRL-2335 and MDA-MB-231 cells treated with AA6 and in KGDH KD cells (Fig. 6g; Suppl. Figure 3h; Suppl. Figure 4h; and Suppl. Figure 7b). Specifically, in 4T1 cells Zeb1 expression was reduced both at mRNA and protein level (Fig. 6f, g) in response to miR-200 family increase as demonstrated by experiments in which miR-200c was KD by validated LNA oligos (Fig. 6h). Moreover, Zeb1 is known to bind CtBP1 corepressor^53,54^, a transcriptional factor associated to cancer progression present among the most modulated genes in the tumour metastasis PCR array analysis reported here and whose down-modulation was validated by qRT-PCR and western blot (Fig. 2a–c). CtBP1 expression down-modulation by AA6 treatment was further confirmed in vitro (Suppl. Figure 7d).

**Fig. 7:**
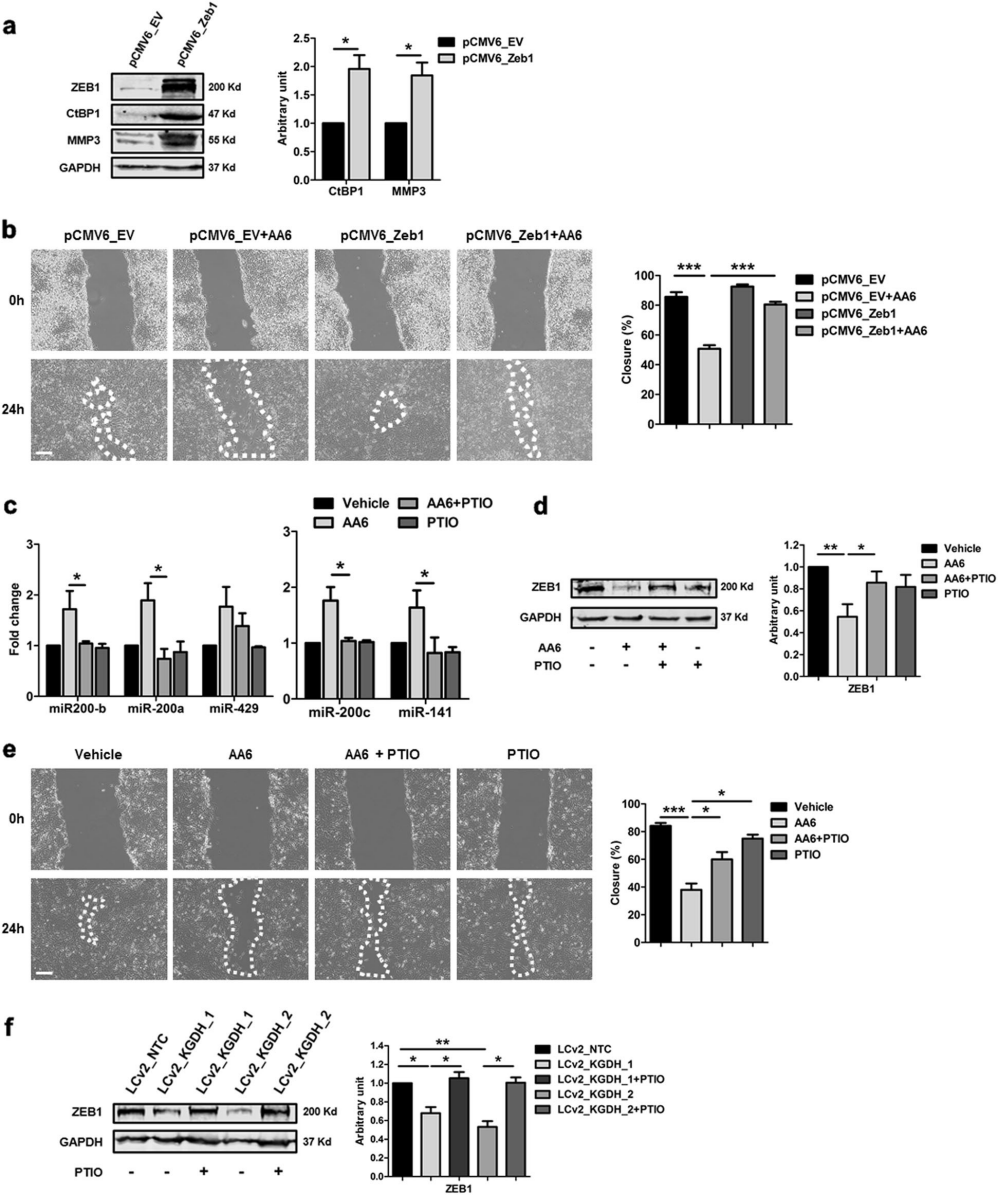
Zeb1 overexpression or PTIO administration counteracts AA6 effect in 4T1 cells. **a** Representative WB (left panel) and relative densitometry (right panel) of ZEB1, CtBP1, and MMP3 protein levels in 4T1 cells transfected with pCMV6_Zeb1 (grey bars) after 48 h compared to control vector (pCMV6; black bars). GAPDH was used as a loading control; *n* = 4. **b** Representative phase contrast images (left panel) depicting 4T1 cells motility and relative percentage of closure measurements (right panel) after Zeb1 overexpression (pCMV6_Zeb1; dark grey bar and pCMV6_Zeb1 + AA6–50 µM; medium grey bar) compared to control vector (pCMV6; black bar and pCMV6 + AA6–50 µM; light grey bars). Scale bar 100 μm; *n* = 4. **c** Cluster 1 (miR-200b, miR-200a, and miR-429; left panel) and cluster 2 (miR-200c and miR-141; right panel) expression in 4T1 cells after 16 h of treatment with vehicle only (black bars), AA6 (50 µM; light grey bars), AA6 (50 µM) + PTIO (100 µM; medium grey bars) and PTIO alone (100 µM; dark grey bars); *n* = 3. **d** Representative WB (left panel) and relative densitometry (right panel) of ZEB1 levels in 4T1 cells after 16 h of treatment with vehicle alone (black bar), AA6 (50 µM; light grey bar), AA6 (50 µM) + PTIO (100 µM; medium grey bar) and PTIO alone (100 µM; dark grey bar). GAPDH was used as a loading control; *n* = 4. **e** Representative phase contrast images (left panel) depicting 4T1 cells motility and relative percentage of closure measurements (right panel) in 4T1 cells after 16 h of treatment with vehicle only (black bar), AA6 (50 µM; light grey bar), AA6 (50 µM) + PTIO (100 µM; medium grey bar) and PTIO alone (100 µM; dark grey bar); *n* = 4. **f** Representative WB (left panel) and relative densitometry (right panel) of ZEB1 levels in 4T1 cells after CRISPR/Cas9 inactivation of KGDH (LCv2_KGDH_1 and LCv2_KGDH_2; light grey bars) compared to control vector (LCv2_NTC; black bar) with or without PTIO (100 µM; dark grey bars) treatment. GAPDH was used as a loading control; *n* = 4. Data are presented as mean ± SE; *p < 0.05, **p < 0.005, ***p < 0.0005 vs controls. Data were analysed by one and two-way ANOVA and non-parametric two-tailed paired Student’s *t*-test

Relevantly, the tumour metastasis PCR array analysis pointed out MMP3 as one of the most down-regulated targets of AA6 (Fig. 2a–c). MMP3 is a matrix-metallo protease contributing to initiate the metastatic spread in various tumours^55^. Recently, Sun et al. demonstrated that MMP3 is an indirect target of miR-200 family/Zeb1 axis^56^ acting on the downregulation of MMP3 *via* Zeb1/phosphorylated-SMAD3 interaction^56^. In our experiments, the anti-metastatic potential of AA6 was further confirmed in vivo and in vitro by the inhibition of MMP3 expression both at mRNA and protein level (Fig. 2b, c; Suppl. Figure 7c, d). Similar effects were observed in 4T1 KGDH KD cells (Suppl. Figure 7a-b). Moreover, to understand whether Zeb1 inhibition was important for AA6 anti-metastatic effect, 4T1 cells were transfected either with an empty vector (pCMV6_EV) or with a plasmid carrying Zeb1 (pCMV6_Zeb1). As expected, transfected cells expressed significantly higher levels of Zeb1 compared to controls (Fig. 7a). The forced expression of Zeb1 was paralleled by an increase in CtBP1 and MMP3 protein expression (Fig. 7a). In this condition, Zeb1 restored the migratory ability of the cells regardless the presence of AA6 (Fig. 7b). Further experiments performed in the presence of the NO scavenger PTIO indicated that AA6-dependent increase of NO played an important role in the miR-200 family response (Fig. 7c) determining Zeb1 down-modulation (Fig. 7d) and motility reduction of 4T1 cells (Fig. 7e). Similar results were obtained in 4T1 KGDH KD cells (Fig. 7f). Taken together these results suggest that, further to TET activation, miR-200 induction, possibly associated with endogenous NO synthesis enhancement, leads to the inhibition of the master EMT inducer Zeb1 conceivably contributing to the anti-metastatic effect of AA6.

## Discussion

The recent evidence that metabolic alteration might contribute to transformation and tumour progression raised interest in anticancer approaches aimed at controlling transformation and metastasization processes^6,12,15^. Exciting results have been obtained in preclinical cancer models where different metabolic pathways have been targeted by selective drugs^7–10^. Specifically, pathways involved in nutrient supplying, energy production and molecular biosynthesis were chosen as novel pharmacological targets^7–10^. Little is known, however, about the potential anticancer effect of drugs able to increase the intracellular level of specific metabolites and their impact on metastasization. In the present study we evaluated whether the novel epi-metabolic drug AA6 might challenge lung metastasis formation in the 4T1 orthotopic mouse model of breast cancer.

4T1 tumour cell line, a recognized model for breast cancer studies originally isolated by Miller and colleagues^57,58^ was exploited. 4T1 cell injection into the mammary gland of BALB/c mice gives rise to a mammary carcinoma with characteristics resembling the human one. After injection, primary tumour grows into the mammary gland and spontaneously develops lung metastasis even after surgical removal of the original tumour, a situation resembling that occurring in real clinical situations^43,59^.

Taking advantage from this model, we found that ketoglutarate dehydrogenase inhibition, following AA6 treatment, reduced the initiating steps of tumour invasion. Indeed, the master regulators of EMT, miR-200 family and Zeb1 transcription factor^37–42^, resulted differentially regulated by KGDH inhibition. Specifically, AA6 induced miR-200 expression and consequently repressed Zeb1 levels. Surprisingly, AA6 not only acted on α-KG levels, but it was also able to increase the expression and nuclear localisation of TET proteins, a fundamental step toward re-activation of DNA demethylation. Indeed, cancer genetic landscape is characterized by hypermethylation paralleled by TET activity alterations^22–24^. Different causes have been accounted for these alterations including (i) specific mutations, (ii) molecular mechanisms leading to reduced expression of TET proteins^25–28^, (iii) delocalisation out of the nucleus^47,48^. In breast cancer, TET activity counteracts tumour progression suppressing metastasis development:^33–35^ it de-represses the expression of tissue metalloproteinase inhibitors^36^ and of anti-metastatic miRNAs, demethylating their promoter regions^35^. In this scenario, the AA6 dependent boost of TET activity, consequent to increased expression and nuclear re-localisation, suggests AA6 as a promising tool to counteract metastasis formation in experimental breast cancer. The inversion between 5mC and 5hmC DNA global levels observed following AA6 treatment, in fact, might specifically hit TET promoter regions activating their transcription. These observations were in line with the recent description that AA6 restored the epi-metabolic control on the DNA demethylation cycle in a metabolically compromised intracellular environment^32^.

KGDH role as AA6 target, was further confirmed by experiments performed in two human breast cancer cell lines (CRL-2335 and MDA-MB-231) and in 4T1 cells in which KGDH was reduced by CRISPR/Cas9 technology. These results pointed out how α-KG fluctuations might interfere with gene transcription regulation. Moreover, the evidence of this communication strengthens the concept of a close interaction between metabolic pathways and epigenetic mechanisms and shed light on the tight cooperation between the cell metabolite factory in mitochondria and the nuclear transcription regulation machinery.

Of interest, our pharmacological approach maintained a residual KGDH activity, possibly important for cellular viability. In fact, it has been clearly established that defects in TCA cycle enzymes, in most of cases, were lethal. Specifically, during early development, KGDH complex knock-out is deleterious determining embryonic lethality in mice^60,61^, profound growth defects in cancer cell lines in vitro and in vivo after xenotransplantation^62^, and α-KG aciduria in human patients with severely decreased levels of KGDH activity^63^. Interestingly, the paucity of patients with KGDH deficiency further support the relevance of this specific enzymatic function^63^. Moreover, these reports pointed out that only KGDH heterozygosity permits normal embryonal development in consequence of residual KGDH activity. Our experimental evidence about KGDH CRISPR/Cas9 cells indeed confirmed that residual KGDH activity is essential for cell viability. For this reason, single clone selection to obtain monoclonal KGDH CRISPR/Cas9 cells was unsuccessful, prompting us to perform experiments in early non-clonal populations obtained after one round of puromycin selection. Hence, we hypothesise that the partial KGDH inhibition could be relevant to the ultimate anticancer effect. The partial KGDH inhibition, in fact, although preserving cellular viability, seemed sufficient to reduce their migration/invasion. Consequently, the anti-metastatic miR-200 family expression was enhanced leading to Zeb1 and CtBP1 expression inhibition.

The present results suggest that KGDH inhibition and the reduction of EMT process are associated with a rapid increase in α-KG. In fact, as depicted in our model (Suppl. Fig. 8), α-KG availability stabilizes TET expression and induces miR-200 promoter demethylation paralleled by an increase of endogenous NO production, fostering their transcription. Interestingly, NO exerts a contradictory effect on metastasis development depending on the primary tumour and the organ target of metastasization^50^. While in some cases NO seems to enhance tumour progression and metastasization^64^, it also hinders aggressiveness of breast cancer^65^ inhibiting cell migration by MMP down-modulation^66^, and lung metastasis reduction^67^. In our context the administration of the epi-metabolic drug AA6, acting on distinct intracellular pathways, counteracted migration, invasion and metastasis development of 4T1 cells.

In conclusion, we provide here compelling evidences supporting AA6 as a promising drug to hinder cancer progression. KGDH targeting led to α-KG increase, able to activate both TET-dependent DNA demethylation and NO production, two molecular/metabolic pathways that might cooperate to hamper the EMT process (Suppl. Fig. 8). Mechanistically AA6, indirectly targeting miR-200 family-Zeb1/CtBP1 axis, decreased Mmp3 expression impairing 4T1 invasion ability in vitro and in vivo. In this view, AA6 might represent a novel epi-metabolically active small molecule useful in the treatment of experimental metastatic breast cancer.

## Materials and methods

### Animal model and treatment

8-week-old female BALB/c mice were purchased from Charles River. To generate spontaneous breast cancer and relative lung metastasis, 10^6^ murine breast cancer 4T1 cells (ATCC® CRL-2539™) were injected into the mammary fat pad in mice under the anaesthesia with isoflurane 2.5%. In all experiments, 4T1 cells were inoculated at day 0. The mice were divided in groups at day 7. The dose and the treatment schedule of (S)-2-[(2,6-dichlorobenzoyl)amino]succinic acid (AA6) were designed as follows: mice received either 50 mg/kg of AA6 (dissolved in PBS, 0.9% p/v NaCl) three times a week or 12.5 mg/kg of AA6 daily, i.p. injections, and were sacrificed after 21 days of treatment (control *n* = 10, 12.5 mg/kg AA6 *n* = 10, 50 mg/kg AA6 *n* = 5). The primary mammary tumours were dissected and measured by digital caliper. The tumour burden in lung was determined by hematoxylin and eosin staining. All animal procedures were approved by the Ethics Committee of the University of Torino, and by the Italian Ministry of Health, in compliance with national and international laws (D.Lgs 26/2014 and Directive 2010/63/EU respectively).

### Cell culture, treatment and transfection

The murine breast cancer 4T1 cells were purchased from ATCC (ATCC® CRL-2539™). Cells were cultured in complete RPMI1640 medium (Gibco) supplemented with 1% L-Glutamine (SIGMA), 1% Penicillin-Streptomycin (SIGMA) and 10% Foetal Bovine Serum (FBS, MILLIPORE). A dose-response curve with the compound AA6 was performed and 50 µM was adopted for further biological evaluations. For the present study, we verified AA6 solubility in water ( > 10 mg/mL) and its stability in cell medium. All subsequent studies were performed using mother solution of AA6 dissolved in water opportunely diluted in cell medium. 4T1 cells were transfected with 1 μg of pCMV6_Zeb1 (OriGene) or empty vector using Lipofectamine 3000 (Invitrogen) according to manufacturer’s instruction. After 48 h, 4T1 cells were collected and used for further analyses. Human umbilical vein endothelial cells (HUVEC) were isolated from human umbilical veins by trypsin treatment (1%) and cultured in M199 medium (SIGMA) with the addition of 20% foetal calf serum (FCS; Gibco), 100 U/mL penicillin (Gibco), 100 μg/mL streptomycin (Gibco), 5 UI/mL heparin, 12 μg/mL bovine brain extract and 200 mM glutamine (Gibco). HUVEC were grown to confluence in flasks and used from the second to the fifth passage. The use of HUVEC was approved by the Ethics Committee of the “Presidio Ospedaliero Martini” of Turin and conducted in accordance with the Declaration of Helsinki. Written informed consent was obtained from all donors. MS-1 from mouse endothelial cells, MDA-MB-231 and CRL2335 cell lines from human breast carcinoma were purchased from ATCC® and cultured in DMEM medium supplemented with 1% L-Glutamine, 1% Penicillin-Streptomycin and 10% FCS.

### Cell motility assay

In the Boyden chamber (BD Biosciences) invasion assay, cells (2 × 10^3^) were plated onto the apical side of 50 µg/mL Matrigel-coated filters (8.2 mm diameter and 0.5 µm pore size; Neuro Probe, Inc.) in serum-free medium with or without increasing concentration of the drugs (0.1–50 μM). Medium containing 20% FCS was placed in the baso-lateral chamber as chemo attractant. After 18 h, cells on the apical side were wiped off with Q-tips. Cells on the bottom of the filter were stained with crystal-violet and counted with an inverted microscope. Data are shown as percentages of migration of treated cells versus the migration measured for vehicle (water) treated cells. Control migration was 72 ± 5 cells (*n* = 5) using MDA-MB-231, 78 ± 6 using CRL2335, and 85 ± 7 for 4T1 (mean ± SE).

### In vitro scratch assay

4T1 cells, KGDH-CRISPR/Cas9 inactivated-4T1 cells and Zeb1-overexpressing 4T1 cells were seeded into 12-well plates and grown to confluence overnight. The cell monolayer was scratched and covered with RPMI1640 medium (Gibco) supplemented with 1% L-Glutamine (SIGMA), 1% Penicillin-Streptomycin (SIGMA), 1% Foetal Bovine Serum (FBS, MILLIPORE) and according to the purpose of experiment treated with or without the drug of interest (AA6: 10, 25 and 50 µM; PTIO: 100 µM). Images were captured after 24 h using a Motic AE2000 light microscope using 10x original magnification (Motic Electric Group Co. Co.). Areas were measured using ImageJ imaging software (BioVoxxel Fiji). For each condition mean was calculated and compared to the area at the starting time point of the experiment.

### Cell adhesion assay

HUVEC or MS-1 were grown to confluence in 24-well plates. Cells were pre-treated or not with increasing concentrations of AA6 (0.1–50 μM) for 1 h, then were co-incubated with human or mouse TNF-α (10 ng/mL) for 18 h and washed twice with fresh medium. Tumour cells (7 × 10^4^ cells/well) were seeded and left to adhere with HUVEC for 1 h. Unattached tumour cells were washed away and the number of adherent cells was evaluated by the Image Pro Plus Software for micro-imaging (Media Cybernetics, version 5.0, Bethesda). Data are shown as percentages of adhesion of AA6-treated cells versus the control adhesion measured on untreated cells; for HUVEC, the control adhesion per microscope field (*n* = 5) was 19 ± 3 using MDA-MB-231, 17 ± 3 using CRL2335; for MS-1, the control adhesion per microscope field (*n* = 12) was 23 ± 4 using 4T1. The TNF-α stimulated adhesion was 193 ± 11% using MDA-MB-231, 231 ± 25 using CRL2335, and 184 ± 14 using 4T1 (mean ± SE).

### Transwell cell invasion assay

The invasiveness of 4T1 cells was measured with a polycarbonate 8-μm porous Transwell membrane (BD Falcon). The top side of the membrane was incubated 1 h at 37° C with 1 mg/mL Matrigel (BD Biosciences) in PBS. 4T1 cells were harvested and re-suspended in RPMI serum free medium at the concentration of 7 × 10^6^ cells/mL. Lower wells of the chamber were loaded with 500 μL RPMI, 2% FBS. Upper wells were loaded with 100 μL cells alone or in the presence of AA6 10, 25 and 50 μM. After incubation for 24 h at 37° C, the top side of the insert membrane was scrubbed free of cells, and the bottom side was fixed with 2.5% glutaraldehyde and stained with 0.1% crystal violet for 15 min, respectively. The images of the invasive cells in the bottom side of the membrane were taken under an Olympus BX60F-3 microscope using a 2.5x original magnification.

### Breast cancer cell viability assay

Cells (2 × 10^3^/well) were seeded in 96-well plates and, after 24 h, treated with different concentrations of AA6 (0.1–50 μM) in complete medium. After 72 h of incubation, viable cells were evaluated by 3-(4,5-Dimethyl-2-thiazolyl)-2,5-diphenyl-2H-tetrazolium bromide, MTT, Methylthiazolyldiphenyl-tetrazolium bromide (MTT, SIGMA) inner salt reagent at 570 nm, as described by the manufacturer’s protocol. The readings from treated cells were expressed as percentage versus control measured on untreated cells.

### Colony-forming assay

Cells (1 × 10^3^/well) were seeded into 6-well plates (well diameter: 34.58 mm) and treated with different concentrations of AA6 (0.1–50 μM) in complete medium. The medium was changed after 72 h and cells were cultured for additional 10 days. Subsequently cells were fixed and stained with a solution of 80% crystal violet and 20% of ethanol. Colonies were then photographed. To induce a completely dissolution of the crystal violet 30% acetic acid was added. Absorbance was detected at 595 nm.

### miR200c-LNA transfection

4T1 cells were transfected with 50 µM Mircury scramble or miR-200c LNA-oligonucleotides (Exiqon) using jetPRIME-siRNA Transfection reagent (Polyplus) according to manufacturer’s instructions. After 16 h, cells were incubated with fresh medium for 32 h and then treated with AA6 or water, as control, for additional 24 h.

### KGDH CRISPR/Cas9 inactivation

To inactivate mouse KGDH, sgRNAs were cloned into LentiCRISPR2 vector (Addgene) using the GoldenGate protocol:^68^

- KGDH_1: Fw 5′-caccgCAGCATCCAAAATCCCCAG-3′;

Rv 5′-aaacCTGGGGATTTTGGATGCTGc-3′;

- KGDH_2: Fw 5′-caccgGTGAACTGCATGATCCCAG-3′;

Rv 5′-aaacCTGGGATCATGCAGTTCACc-3′;

Specific CRISPR/Cas9 transfection was compared to a non-targeting control (NTC) sgRNA:

- NTC: Fw 5′-caccgTTCCGGGCTAACAAGTCCT-3′;

Rv 5′-aaacAGGACTTGTTAGCCCGGAAc-3′.

The obtained plasmids were transformed into NEB 5-alpha Competent *E. coli* (High Efficiency –New England Biolabs), then DNA was purified by EZNA Fastfilter Endo-Free Plasmid DNA Maxi Kit (Omega Bio-Tek), and a concentration of 6 μg was used for transfection in 4T1 cells; the transfection was performed using Lipofectamine 3000 (Invitrogen) according to the manufacturer’s protocol. All experiments were performed by using the polyclonal population emerging after a round of puromycin selection showing residual KGDH expression, specifically, after 48 h 4T1 cells were selected by 1.5 μg/mL puromycin recovered from selection and tested for KGDH knock-out by western blot.

### Enzymatic activity assay and metabolite quantification

KGDH Activity Assay Colorimetric Kit (K678, BioVision), TET Activity/Inhibition Assay Colorimetric Kit (P3086, Epigentek), the α-KG Assay Colorimetric/Fluorometric Kit (K677, BioVision), and Arginine quantification kit (CEB938Ge, Cloud-Clone Corp.) were performed according to manufacturer’s instructions. Signals were detected by EnSpire Multimode Plate Reader (Perkin Elmer).

### Nitric oxide quantification

Nitrate/Nitrite Colorimetric Assay Kit (780001, Cayman Chemical) was performed according to manufacturer’s instructions. AA6-treated/untreated 4T1-injected mice tumorigenic tissue samples (25 mg) were lysed in RIPA buffer (Tris HCl pH 7.4 10 mM, NaCl 150 mM, NP-40 1%, sodium deoxycholate (DOC) 1%, SDS 0.1%, glycerol 0.1%, Protease Inhibitors Cocktail), homogenized and centrifuged, 200 µg of freshly lysed sample were used per condition. The NO-final products NO_3_^−^ + NO_2_^−^ were colorimetrically detected by EnSpire Multimode Plate Reader (Perkin Elmer). In vitro NO production was evaluated by adding 4,5-diaminofluorescein diacetate (DAF-2DA, Cayman Chemical) according to manufacturer’s instructions to 4T1 cultured 3, 6, 16, 24 h with 50 µM AA6 or vehicle alone, to 4T1 cells after 16 h of treatment with vehicle only (water), AA6 (50 µM) and AA6 (50 µM) + PTIO (SIGMA, 100 µM) and to 4T1 cells after CRISPR/Cas9 inactivation of KGDH ± PTIO (100 µM). At the end of treatment, cells were collected and analyzed by FACS (FACS Canto II-BD) to detect intracellular NO production.

### 5-methylcytosine and 5-hydroxymethylcytosine global level quantification

Genomic DNA extraction from AA6-treated/untreated 4T1-injected mice tumorigenic tissue (25 mg) and AA6-treated/untreated 4T1 cells was performed using the E.Z.N.A. Tissue DNA kit (D3396, Omega Bio-Tek). 5mC and 5hmC global levels were evaluated using the ELISA-based MethylFlash Methylated DNA Quantification Colorimetric Kit (P1034, Epigentek) and the MethylFlash Hydroxymethylated DNA Quantification Colorimetric Kit (P1036, Epigentek) respectively. The optical density (OD) was detected by EnSpire Multimode Plate Reader (Perkin Elmer).

### Detection of 5mC on miR-200 family promoter

The 5mC enrichment on miR-200 family promoter was analyzed by EpimarK 5mC and 5hmC Analysis Kit (New England Biolabs) according to manufacturer’s instructions. Briefly, DNA was isolated from AA6-treated/untreated 4T1-injected mice tumorigenic tissue (25 mg) using the E.Z.N.A. Tissue DNA kit (D3396, Omega Bio-Tek). The amplified regions were selected on the bases of CCpGG sites reported on MethPrimer 2.

The following primers were used:

Cluster1 r1 F: 5′- TTTCTATCACAGACACAATACAG -3′

Cluster1 r1 R: 5′- GAAGTATATCTGACGGGTGT -3′

Cluster1 r2 F: 5′- GGTAGCCTGAGTGTAGACAAGACA -3′

Cluster1 r2 R: 5′- CTCTGCAGCAAGCACCCTCC-3′

Cluster2 r1 F: 5′- AAGGAGGAAGAGCGAGAGTG -3′

Cluster2 r1 R: 5′- CCATTTACTGCGTTCTACCGT -3′

Cluster2 r2 F: 5′- TGTTTGGGTGCTGGTTGGGA -3′

Cluster2 r2 R: 5′- CCACCCTTAACTCGGAAGAAG -3′

### RNA Extraction, RT-PCR and miRNA Analysis

Total RNA was isolated either from samples deriving from AA6-treated/untreated 4T1 cells or from AA6-treated/untreated 4T1-injected mice tumorigenic tissue (10–25 mg) using Tri-Reagent (SIGMA) according to the instructions of the manufacturer. cDNA synthesis for quantitative real-time PCR (qRT-PCR) was carried out with qScript cDNA SuperMix (95048, Quanta BIOSCIENCES) following the manufacturer’s protocol. All reactions were performed in 96-well format in the StepOne Plus Real-Time PCR System (Applied Biosystems) using PerfeCTa SYBRGreen FastMix, ROX (Quanta BIOSCIENCES). Each RNA sample was tested in duplicate and P0 was used as housekeeping gene. The Applied Biosystem software’s Comparative Ct Method (Applied Biosystem) was used to calculate mRNA expression levels, data were presented as fold change of transcripts for target genes. Fold change below 1 shows downregulated expression versus controls. Primer sequences used for mRNA analysis were selected based on published sequence data from NCBI database and listed below:

**Table:** 

Gene	Species	Forward (5′–3′)	Reverse (5′–3′)
ZEB1	Mus Musculus	AGACCAGACAGTATTACCAG	CAGAAATTCTTCCACATT
MMP3	Mus Musculus	ACATGGAGACTTTGTCCCTTTTG	TTGGCTGAGTGGTAGAGTCCC
GPNMB	Mus Musculus	AGAAATGGAGCTTTGTCTACGTC	CTTCGAGATGGGAATGTATGCC
CtBP1	Mus Musculus	CAAGAAGGAAGTCAGCCCAG	GCCTCAATGAGCACAACCAC
PLAUR	Mus Musculus	CAGAGCTTTCCACCGAATGG	GTCCCCGGCAGTTGATGAG
SRC	Mus Musculus	GCTAGAGGCTGGTGTTGATTG	GAGTCTGCTGGACTTTCTC
TET1	Mus Musculus	GAAGGAACAGGAAGCTGCAC	CTGGCCAAACCTAGTCTCCA
TET2	Mus Musculus	GATCCAGGAGGAGCAGTGAG	TGGGAGAAGGTGGTGCTATC
TET3	Mus Musculus	CCGGATTGAGAAGGTCATCTAC	AAGATAACAATCACGGCGTTCT
P0	Mus Musculus	GCGTCCTGGCATTGTCTGT	GAAGGCCTTGACCTTTTCAGTAAG

Primers for miR-16, miR-141, miR-200a, miR-200b, miR-200c, miR-429, primiR-200 cluster1, primiR-200 cluster2 and the reagents for reverse transcriptase and qPCR reactions were all obtained from Applied Biosystems. Relative expression was calculated using the comparative cycle threshold (Ct) method (ΔΔCt). miRNA expression levels in each sample were normalised to miR-16 expression as, under the experimental conditions of the present study, miR-16 was not modulated by AA6 treatment.

### Metastasis RT^2^ profiler PCR array

RNA was extracted from AA6-treated/untreated 4T1-injected mice tumorigenic tissue (10–25 mg) using Tri-Reagent (SIGMA) according to supplier’s instructions. Briefly, total mRNA was treated with column DNase treatment (QIAGEN RNeasy mini kit) and converted to cDNA (RT^2^ First Standard Kit, 330401, QIAGEN). cDNA samples were mixed with the ready-to-use RT^2^ SYBR Green ROX qPCR master mix (330522, QIAGEN), and equal volumes were aliquoted to each well of the same plate (RT^2^ Profiler PAMM028ZC, QIAGEN) to perform the real-time PCR cycling programme.

### Western blotting

Western blot analyses were performed according to standard procedures. AA6-treated/untreated 4T1-injected mice tumorigenic tissue (10–25 mg) and AA6-treated/untreated 4T1 cells samples were lysed in Laemmli buffer (Tris HCl 100 mM pH 6.8, SDS 4%, glycerol 20%, DTT 25 mM, NuPAGE LDS Sample Buffer 1x - Invitrogen). Nitrocellulose blotting membranes were probed with the following antibodies: ZEB-1 (Santa Cruz), CtBP-1 (Cell Signaling), GPNMB (Thermo Fisher Sc.), MMP-3 (BIOSS), SRC (Cell Signaling), KGDH (alias OGDH, Genetex), TET-1 (Genetex), TET-2 (Santa Cruz) and TET-3 (Novus Biologicals) flag (SIGMA), α-tubulin (Cell Signaling), GAPDH (abcam), Grb2 (Santa Cruz). Signals were detected by Odyssey CLx Infrared Imaging System (LI-COR Biosciences). Optical density values of specific proteins were normalized to that of tubulin and corrected for those obtained from controls that were considered equal to 1.

### Subcellular fractionation

5 × 10^6^ AA6-treated/untreated 4T1 cells were lysed with the lysis buffer provided by Qproteome Cell Compartment kit (QIAGEN), then fractionation was performed according to the manufacturer’s instructions. Subcellular fraction content was normalised according to Comassie staining before western blotting.

### Immunofluorescence and confocal microscopy

Confocal analysis was performed according to standard procedures in AA6-treated/untreated 4T1 cells fixed with 4% paraformaldehyde. TET-1 (1:150, monoclonal, Genetex), TET-2 (1:100, polyclonal, Santa Cruz) and TET-3 (1:150, polyclonal, Novus Biologicals) antibodies were used. Samples were analysed using a Leica TCS SP8 confocal microscope using a 40x original magnification. Confocal analysis of primary tumours was performed to standard procedures in AA6-treated/untreated primary tumours using Ki67 (1:400, monoclonal, ThermoFisher Scientific), Cleaved Caspase-3 (1:50, monoclonal, Cell Signaling) antibodies. Immunofluorescence images were captured by utilising a Leica TCS SPE confocal laser-scanning microscope using a 20x original magnification (Leica Microsystems) and by maintaining the same laser power, gain and offset settings. All immune-localisation experiments were performed on multiple tissue sections.

### Immunohistrochemistry

Immunihostochemistry was performed with enzymatic induced epitope retrieval procedures in AA6-treated/untreated lungs, using Ki67 (1:200, monoclonal ThermoFisher Scientific) and Cleaved Caspase-3 (1:50, monoclonal, Cell Signaling). The images were taken under an Olympus BX60F-3 microscope using a 20x and a 40x original magnification.

### Statistical analysis

Non parametric student’s t-test was used to analyse variables. Significance between experimental groups was determined by one or two-way ANOVA followed by the Bonferroni’s multiple comparison post tests using GraphPad InStat software. For tumour volume and lung metastasis we applied non-parametric Mann Withney test while for the incidence of lung metastasis we applied Mantel-Cox Test. Overall values of *p* ≤ 0.05 were deemed statistically significant. Data indicate the mean values of at least three independent experiments ± SE. A specific comment has been added for each analysis.

## Electronic supplementary material

Supplemental Figures

Supplemental table
