# Supplementary material for: α-ketoglutarate dehydrogenase inhibition counteracts breast cancer-associated lung metastasis
Source: Cell Death Dis. 2018 Jul 9;9(7):756. doi: 10.1038/s41419-018-0802-8 (PMC6037705; doi:10.1038/s41419-018-0802-8)
Supplement: Supplementary file 1 — Supplemental Figures [file 41419_2018_802_MOESM1_ESM.docx]

**Supplemental figures**

**
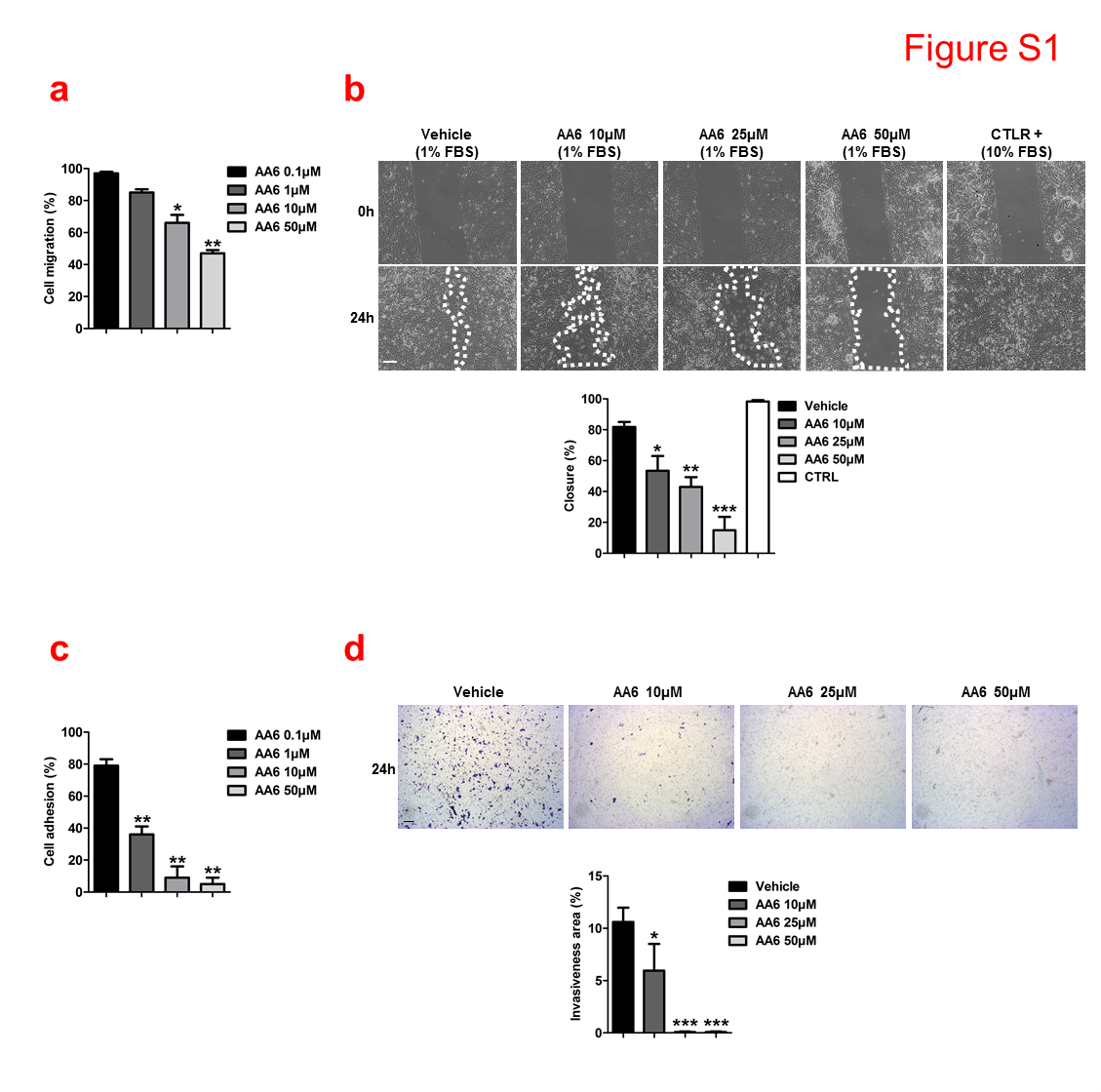
**

**S1. AA6 dose-dependently impairs 4T1 cells migration, adhesion to endothelium and invasion capacity (a)** Graph showing 4T1 cell migration after 18 h treatment with AA6 at different concentrations (0.1 - 1 - 10 - 50 µM) as percentages versus vehicle treated cells; medium containing 20% FCS was used as chemo attractant; n = 5. **(b)** Representative phase contrast microscopy images (upper panel) depicting 4T1 cells motility after 24 h treatment with AA6 at different concentrations (10 - 25 - 50 µM) or vehicle alone; the graph (lower panel) shows the percentage of closure in 4T1 cells after 24 h treatment with AA6 (grey bars) or vehicle only (black bar); 20% FCS was used as control. Scale bar 100 μm; n = 5 each group. **(c)** Graph showing 4T1 cells adhesion to MS-1 after 24 h treatment with AA6 at different concentrations (0.1 - 1 - 10 - 50 µM) as percentages versus the control adhesion measured in TNF-α treated cells taken as 100%; n = 5. **(d)** Representative pictures (upper panel) showing 4T1 cells invasiveness after AA6 treatment at different concentrations (10 - 25 - 50 µM) versus vehicle alone; the graph (lower panel) represents migrated cells counted after 24 h treatment with AA6 (grey bars) or vehicle only (black bar). Scale bar 50 μm; n = 3. Data are presented as means ± SE; *p < 0.05, **p < 0.005, ***p < 0.0005 vs control. Data were analyzed by one-way ANOVA.


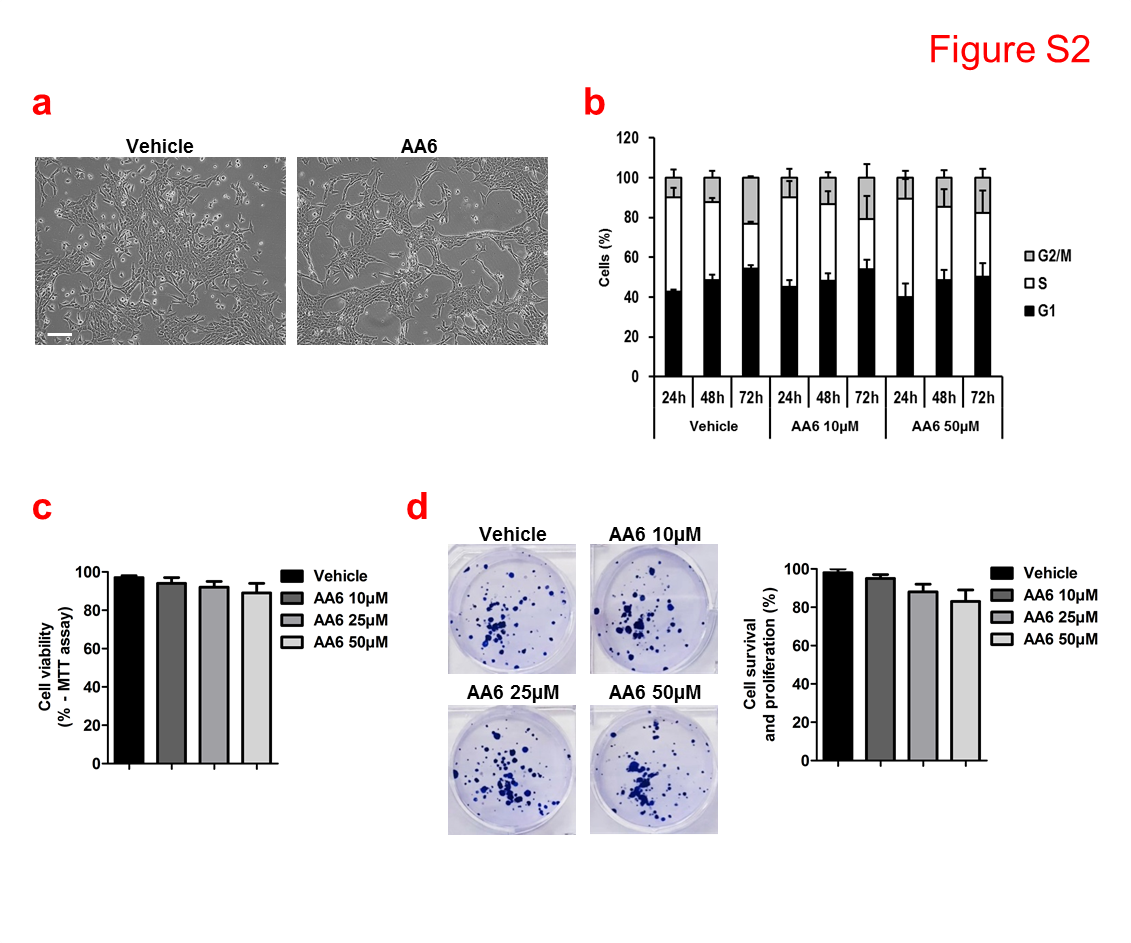


**Figure S2.** **AA6 administration does not interfere with 4T1 cells** **survival, cell cycle, and proliferation (a)** Representative phase contrast microscopy images depicting 4T1 cells proliferation after 24 h treatment with AA6 (50 µM) or vehicle alone. Scale bar 100 μm. **(b)** Bar graph showing the percentages for G1, S, and G2/M at 24 - 48 -72 h in 4T1 cells treated with AA6 (10 - 50 µM) or vehicle only, then pulse-labelled with BrdU for 30 min before harvesting, stained with Propidium iodide (PI) and subjected to flow cytometry analysis for their DNA content; n=3. **(c)** Relative percentage of 4T1 cells viability after treatment with AA6 (10 - 25 - 50 µM) or vehicle alone, determined by MTT assay (Sigma) after 72h; n=8 each group. **(d)** Representative images (left panel) showing 4T1 cells colony formation determined by clonogenic assay after treatment with AA6 (10 - 25 - 50 µM) or vehicle only; cells were fixed and stained by crystal violet dissolved in 30% acetic acid. The graph (right panel) represents cell survival and proliferation at 10 days after treatment with AA6 (grey bars) or vehicle alone (black bar). Well diameter 34.58 mm; n = 5. Data are presented as means ± SE vs control. Data were analyzed by one and two-way ANOVA.


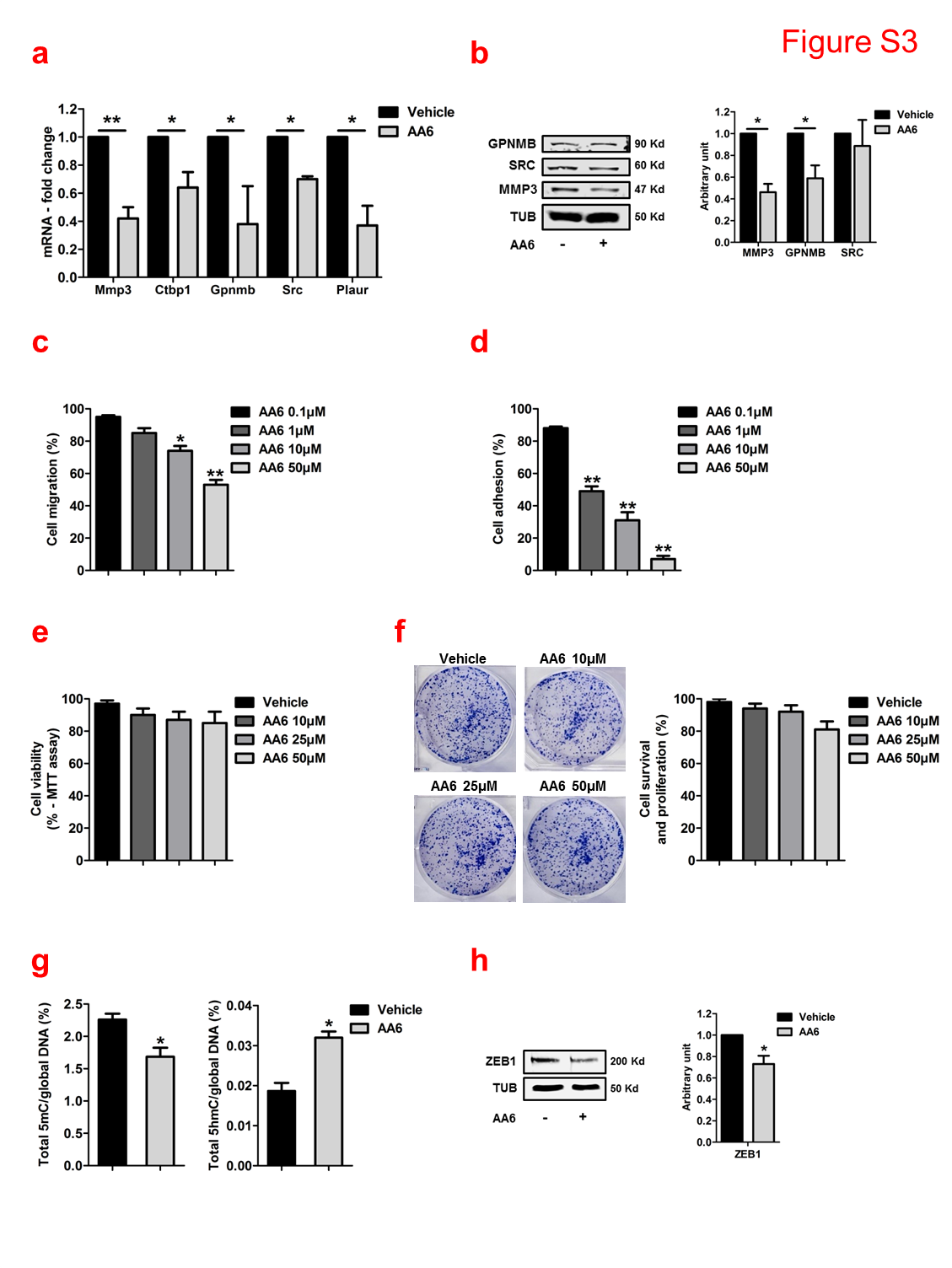


**Figure S3.** **AA6 administration does not interfere with CRL2335 cells viability, modulates 5mC/5hmC global levels and impairs CRL2335 cells migration and adhesion acting along the Zeb1/CtBP1–MMP3 axis (a)** mRNA expression analysis of Mmp3, Ctbp1, Gpnmb, Src and Plaur genes in CRL2335cells after 48 h of AA6 administration (50 µM; grey bars) compared to vehicle alone (black bars); n = 4. **(b)** Representative western blot (right panel) and relative densitometry (left panel) of MMP3, GPNMB and SRC protein levels in CRL2335 cells after 48 h of AA6 administration (50 µM; grey bars) compared to vehicle alone (black bars). α-tubulin was used as loading control; n = 4. **(c)** Graph showing CRL2335 cell migration after 18 h treatment with AA6 at different concentrations (0.1 - 1 - 10 - 50 µM) as percentages versus vehicle-treated cells; medium containing 20% FCS was used as chemo attractant; n = 5. **(d)** Graph showing CRL2335 cells adhesion after 24 h treatment with AA6 at different concentrations (0.1 - 1 - 10 - 50 µM) as percentages versus the control adhesion measured in TNF-α treated cells taken as 100%; n = 5. **(e)** Relative percentage of CRL2335 cells viability after treatment with AA6 (10 - 25 - 50 µM), compared to vehicle-treated cells, determined by MTT assay (Sigma) after 72h; n = 8 each group. **(f)** Representative images (left panel) showing CRL2335 cells colony formation determined by clonogenic assay after treatment with AA6 (10 - 25 - 50 µM) compared to vehicle only; cells were fixed and stained by crystal violet dissolved in 30% acetic acid. The graph (right panel) represent cell survival and proliferation at 10 days after treatment with AA6 (grey bars) or vehicle alone (black bar). Well diameter 34.58 mm; n = 5. **(g)** Quantification of 5mC (left panel) and 5hmC (right panel) global levels in CRL2335 cells exposed to AA6 (50 µM; grey bars) for 48 h indicated as fold-change versus vehicle-treated cells (black bars); n = 3 each group. **(h)** Representative western blot (right panel) and relative densitometry (left panel) of ZEB1 protein levels in CRL2335 cells after 48 h of AA6 administration (50 µM; grey bar) compared to vehicle (black bar). α-tubulin was used as loading control; n = 4. Data are presented as means ± SE; *p < 0.05, **p < 0.005 vs control. Data were analyzed by one-way ANOVA and two-tailed paired Student's t-test.


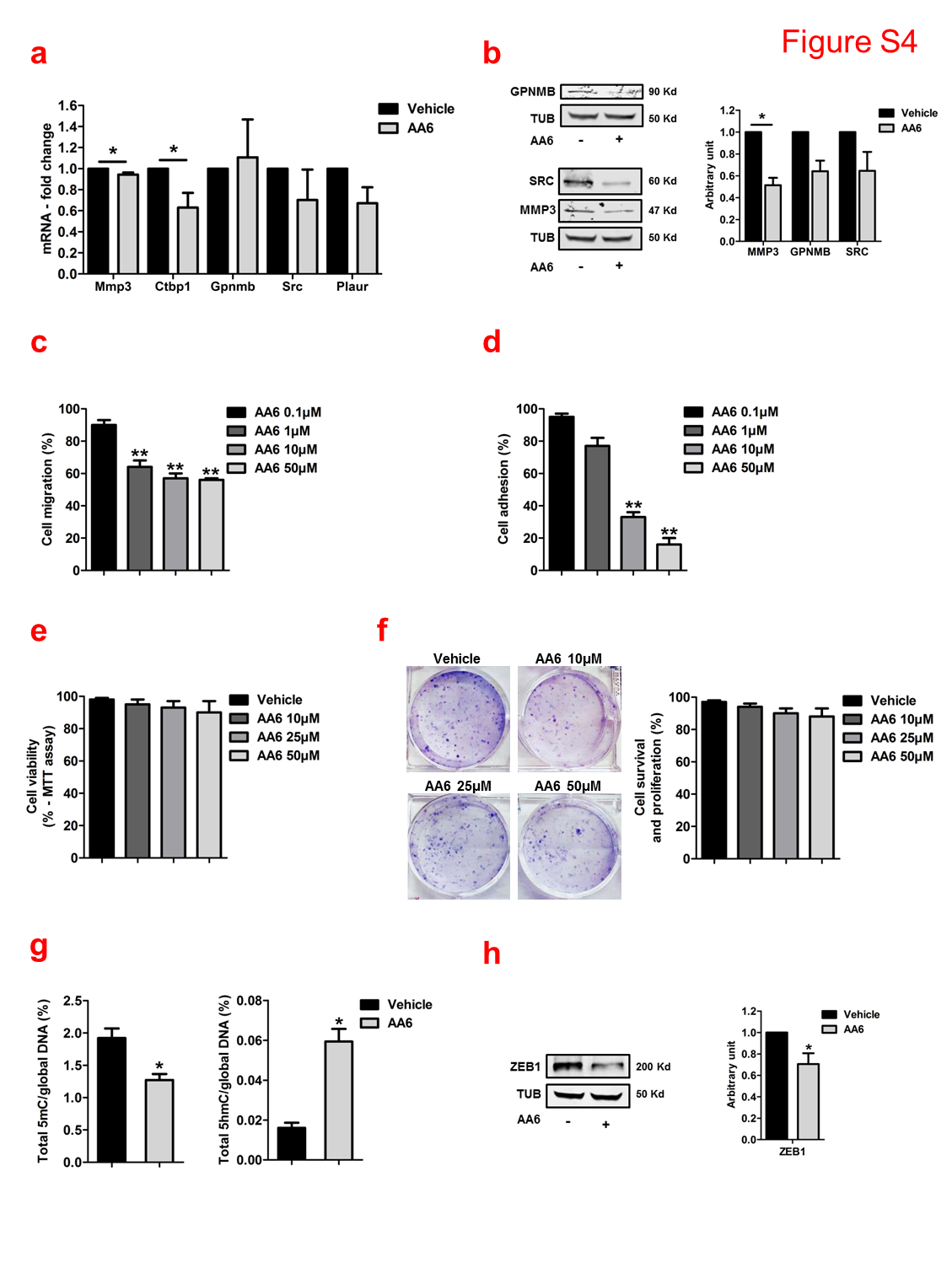


**Figure S4.** **AA6 administration does not interfere with MDA-MB-231 cells viability, modulates 5mC/5hmC global levels and impairs MDA-MB-231 cells migration and adhesion acting along the Zeb1/CtBP1–MMP3 axis (a)** mRNA expression analysis of Mmp3, Ctbp1, Gpnmb, Src and Plaur genes in MDA-MB-231 cells after 48 h of AA6 administration (50 µM; grey bars) compared to vehicle alone (black bars); n = 4. **(b)** Representative western blots (right panels) and relative densitometry (left panel) of MMP3, GPNMB and SRC protein levels in MDA-MB-231 cells after 48 h of AA6 administration (50 µM; grey bars) compared to vehicle alone (black bars). α-tubulin was used as loading control; n = 4. **(c)** Graph showing MDA-MB-231 cell migration after 18 h treatment with AA6 at different concentrations (0.1 - 1 - 10 - 50 µM) as percentages versus vehicle-treated cells; medium containing 20% FCS was used as chemo attractant; n = 5. **(d)** Graph showing MDA-MB-231 cells adhesion after 24 h treatment with AA6 at different concentrations (0.1 - 1 - 10 - 50 µM) as percentages versus the control adhesion measured in TNF-α treated cells taken as 100%; n = 5. **(e)** Relative percentage of MDA-MB-231 cells viability after treatment with AA6 (10 - 25 - 50 µM) compared to vehicle-treated cells, determined by MTT assay (Sigma) after 72 h; n = 8 each group. **(f)** Representative images (left panel) showing MDA-MB-231 cells colony formation determined by clonogenic assay after treatment with AA6 (10 - 25 - 50 µM) or vehicle only; cells were fixed and stained by crystal violet dissolved in 30% acetic acid. The graph (right panel) represent cell survival and proliferation at 10 days after treatment with AA6 (grey bars) or vehicle alone (black bar). Well diameter 34.58 mm; n = 5. **(g)** Quantification of 5mC (left panel) and 5hmC (right panel) global levels in MDA-MB-231 cells exposed to AA6 (50 µM; grey bars) for 48 h indicated as fold-change versus vehicle-treated cells (black bars); n = 3 each group. **(h)** Representative western blot (right panel) and relative densitometry (left panel) of ZEB1 protein levels in MDA-MB-231 cells after 48 h of AA6 administration (50 µM; grey bar) compared to vehicle (black bar). α-tubulin was used as loading control; n = 4. Data are presented as means ± SE; *p < 0.05, **p < 0.005 vs control. Data were analyzed by one-way ANOVA and two-tailed paired Student's t-test.

**
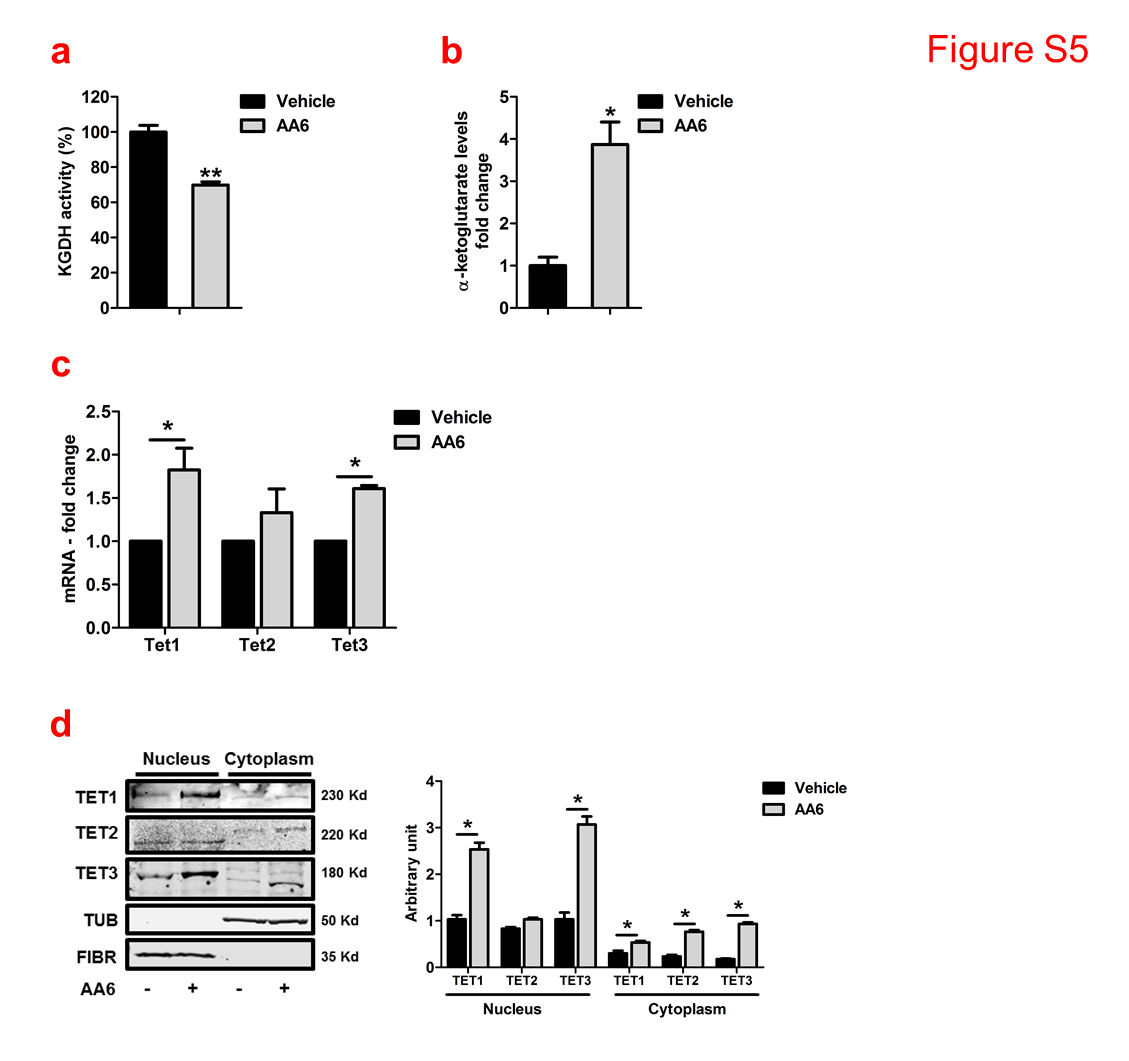
**

**Figure S5.** **KGDH inhibition increases TETs expression *in vitro*** **(a)** KGDH activity and **(b)** α-KG level quantification in AA6 (50 µM; grey bars) treated 4T1 cells compared to vehicle (black bars) after 16 h; n = 3 each group. **(c)** Tet1, 2, 3 mRNA expression levels in AA6 (50 µM; grey bars) treated 4T1 cells compared to vehicle (black bars) after 16 h; n = 3 each group. **(d)** Representative western blot (right panel) and relative densitometry (left panel) depicting the subcellular localization of TET1, 2, 3 in AA6 (50 µM; grey bars) treated 4T1 cells compared to vehicle-treated cells (black bars) after 16 h; n = 3. Fibrillin (FIBR) and α-tubulin (TUB) were used as loading controls for nuclei and cytoplasms respectively. Data are presented as mean ± SE; *p < 0.05, **p < 0.005 vs control. Data were analyzed by one-way ANOVA and non-parametric two-tailed paired Student's t-test.


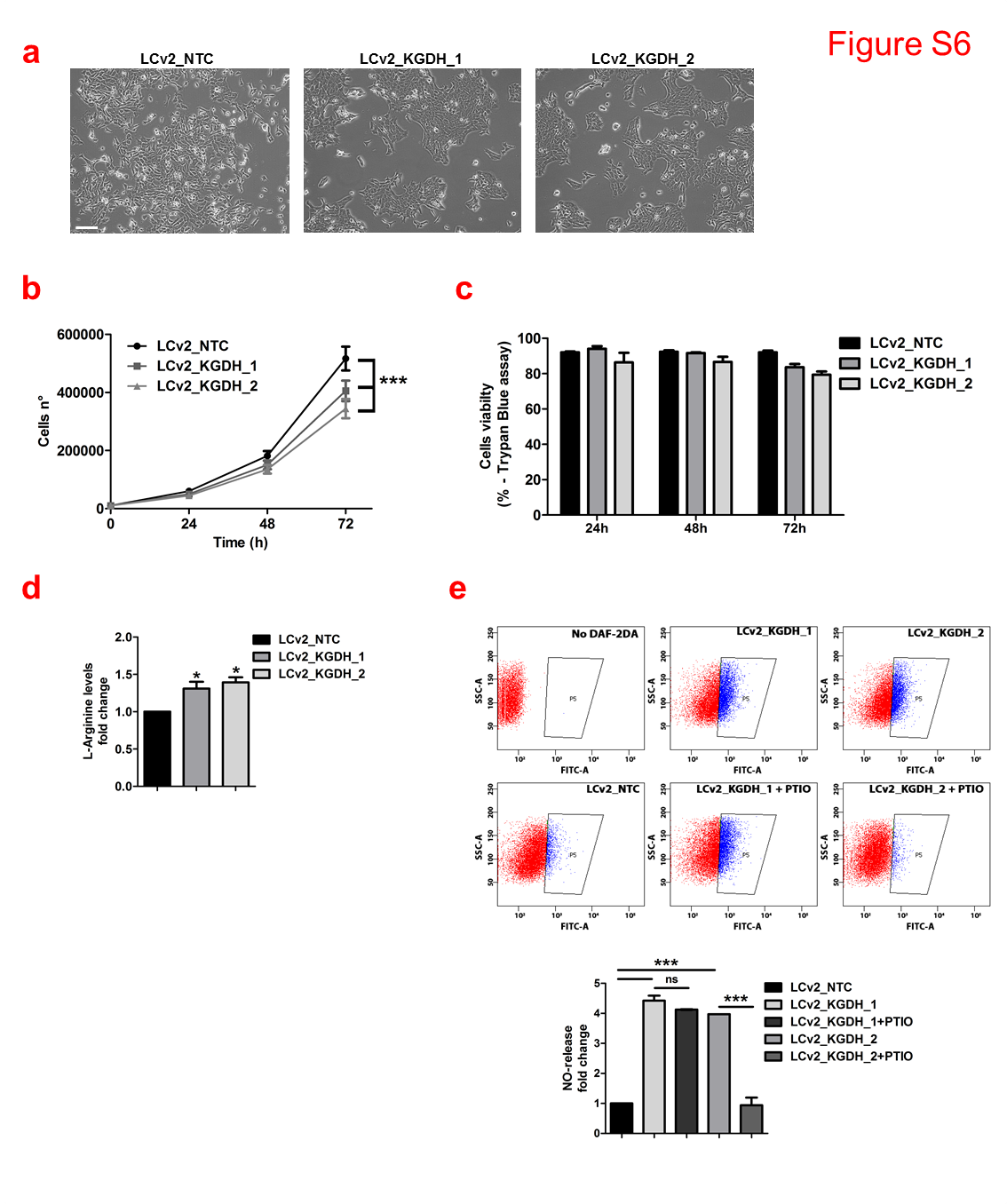


**Figure S6.** **CRISPR/Cas9** **KGDH partial inactivation does not interfere with 4T1 cells viability and increases nitric oxide-release (a)** Representative phase contrast microscopy images depicting 4T1 cells proliferation after CRISPR/Cas9 inactivation of KGDH (LCv2_KGDH_1 and LCv2_KGDH_2) compared to control vector (LCv2_NTC). Scale bar 100 μm. **(b)** Growth curve showing cells proliferation rate in 4T1 cells after CRISPR/Cas9 inactivation of KGDH (LCv2_KGDH_1 and LCv2_KGDH_2; grey lanes) over 72 h compared to control vector (LCv2_NTC; black lane); n = 3 each group. **(c)** Relative percentage of cells viability in 4T1 cells after CRISPR/Cas9 inactivation of KGDH (LCv2_KGDH_1 and LCv2_KGDH_2) compared to control vector (LCv2_NTC) at 24 - 48 -72 h, detected by trypan blue staining; n = 3 each group. **(d)** L-Arginine quantification in 4T1 cells after CRISPR/Cas9 inactivation of KGDH (LCv2_KGDH_1 and LCv2_KGDH_2; grey bars) compared to control vector (LCv2_NTC; black bars); n = 3. **(e)** Representative dot plot (upper panel) and relative quantification (lower panel) showing NO-release evaluation by FACS analysis of DAF-2 DA stained 4T1 cells after CRISPR/Cas9 inactivation of KGDH (LCv2_KGDH_1 and LCv2_KGDH_2; light grey bars) ± PTIO (100 µM; dark grey bars) compared to control vector (LCv2_NTC; black bar); n = 3. Data are presented as mean ± SE; *p < 0.05, ***p < 0.0005 vs control. Data were analyzed by one-way ANOVA and non-parametric two-tailed paired Student's t-test.


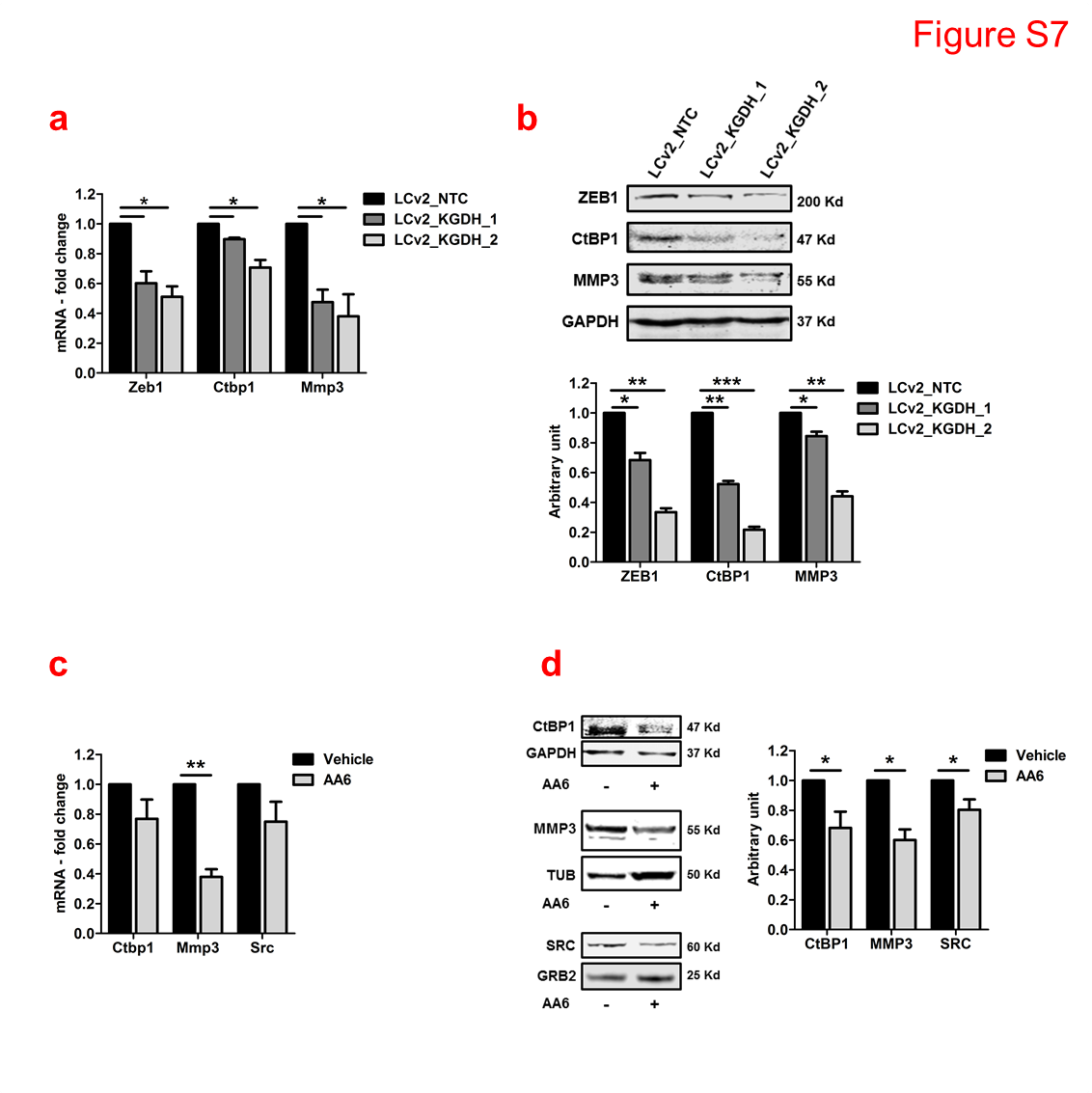


**Figure S7.** **AA6 acts along the TET–miR200–Zeb1/CtBP1–MMP3 axis in 4T1 cells. (a)** Zeb1, Ctbp1 and Mmp3 mRNA expression in 4T1 cells after CRISPR/Cas9 inactivation of KGDH (LCv2_KGDH_1 and LCv2_KGDH_2; grey bars) indicated as fold-change versus control vector (LCv2_NTC; black bar); n = 3. **(b)** Representative WB (upper panel) and relative densitometry (lower panel) of ZEB1, CtBP1 and MMP3 protein levels in 4T1 cells after CRISPR/Cas9 inactivation of KGDH (LCv2_KGDH_1 and LCv2_KGDH_2) compared to control vector (LCv2_NTC). GAPDH was used as loading control; n = 3. **(c)** Ctbp1, Mmp3 and Src mRNA expression in 4T1 cells after 48 h of AA6 administration (50 µM; grey bars) compared to vehicle alone (black bars); n = 4. **(d)** Representative WB (left panels) and relative densitometry (right panel) of CtBP1, MMP3 and SRC in 4T1 cells after 48 h of AA6 administration (50 µM; grey bars) compared to vehicle alone (black bars). α-tubulin, GAPDH and GRB2 were used as loading controls; n = 4. Data are presented as mean ± SE; *p < 0.05, **p < 0.005, ***p < 0.0005 vs **
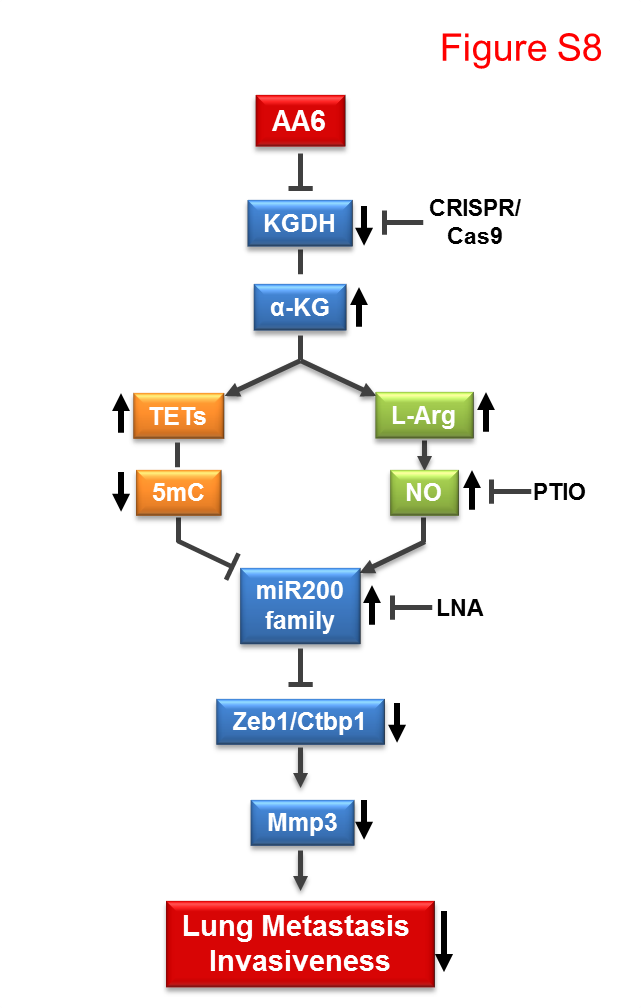
**controls. Data were analyzed by non-parametric two-tailed paired Student's t-test.

**Figure S8. Schematic representation: AA6 acts along TET–miR200–Zeb1/CtBP1–MMP3 axis to prevent lung metastasis development** AA6 inhibits KGDH increasing α-KG synthesis both *in vitro* and *in vivo*. The α-KG enhances TET activity, leading to DNA demethylation and favoring endogenous NO production via L-Arginine synthesis. Both mechanisms converge to induce miR-200 family expression and consequently to down-modulate Zeb1 and Ctbp1 as well as Mmp3. The shut-down of this molecular pathway interferes with the EMT process and lung metastasis progression.
