# Supplementary material for: α-ketoglutarate dehydrogenase inhibition counteracts breast cancer-associated lung metastasis
Source: Cell Death Dis. 2018 Jul 9;9(7):756. doi: 10.1038/s41419-018-0802-8 (PMC6037705; doi:10.1038/s41419-018-0802-8)
Supplement: Supplementary file 2 — Supplemental table [file 41419_2018_802_MOESM2_ESM.pdf]

Supplemental Table 1. Metastasis associated genes regulated by AA6 treatment compared to saline control depicted in Figure 2a.

	Fold change	p value
Fat1	0,546	0,002
Gpnmb	0,284	0,007
Tcf20	0,705	0,010
Src	0,409	0,014
Met	2,193	0,018
Ctbp1	0,335	0,018
Il18	1,792	0,020
Brms1	0,768	0,025
Apc	2,242	0,030
Lpar6	2,216	0,045
Mmp3	0,328	0,050
Cdh11	3,658	0,064
Smad2	0,734	0,078
Plaur	0,473	0,078
Tnfsf10	3,752	0,111
Fn1	1,605	0,119
Tgfb1	0,723	0,148
Hras	0,770	0,154
Ewsr1	0,732	0,156
Col4a2	0,704	0,176
Cd44	0,698	0,179
Myc	1,541	0,181
Pnn	1,170	0,194
Vegfa	0,480	0,205
Cdh6	0,376	0,209
Hgf	1,920	0,234
Nme2	1,176	0,256
Nf2	0,854	0,320
Htatip2	0,747	0,340
Cd82	1,158	0,341
Csf1	0,805	0,397
Cxcr2	0,610	0,420
Mdm2	1,162	0,445
Mmp7	0,638	0,451
Il1b	1,079	0,475
Mta1	1,127	0,482
Itgb3	0,898	0,487
Mmp13	1,065	0,489
Nme1	0,875	0,518
Trp53	0,960	0,584
Ctsl	0,739	0,585
Smad4	1,082	0,666
Fgfr4	1,295	0,668
Nr4a3	1,193	0,676
Etv4	1,140	0,680
Ctnna1	1,094	0,693
Mmp10	1,031	0,714

Mtss1	0,943	0,798
Rpsa	1,066	0,838
Syk	1,177	0,915
Chd4	1,089	0,929
Mmp9	1,379	0,938
Timp2	0,991	0,954
